# Design, Synthesis, and Biological Evaluation of Trifluoromethylated 1,2,4-Triazin-6-ones with Selective Antimycotic Activity and Hormesis Effects

**DOI:** 10.3390/ijms27156796

**Published:** 2026-07-29

**Authors:** Anna Kowalczyk, Aleksandra Trębska, Milena Sęczkowska, Katarzyna Gach-Janczak, Anita Ciesielska, Paweł Stączek, Marcin Jasiński

**Affiliations:** 1 Department of Organic and Applied Chemistry, Faculty of Chemistry, University of Lodz, Tamka 12, 91-403 Łódź, Poland; annakowalczyk1312@gmail.com (A.K.); milena.seczkowska@edu.uni.lodz.pl (M.S.); 2 Department of Molecular Microbiology, Faculty of Biology and Environmental Protection, University of Lodz, Banacha 12/16, 90-237 Łódź, Polandanita.ciesielska@biol.uni.lodz.pl (A.C.); pawel.staczek@biol.uni.lodz.pl (P.S.); 3 Department of Biomolecular Chemistry, Medical University of Lodz, Mazowiecka 6/8, 92-215 Łódź, Poland; katarzyna.gach@umed.lodz.pl

**Keywords:** fluoroorganics, nitrile imines, synthesis, (3+3)-cycloadditions, 1,2,4-triazines, antimycotics, selectivity, hormesis, antimicrobial study, cytotoxicity analysis

## Abstract

The emergence of antifungal resistance highlights the need for new chemotypes with improved biological activity. In this study, a series of new 3-CF_3_-1,2,4-triazin-6(1*H*)-ones was synthesized via a (3+3)-cycloaddition of in situ-generated CF_3_-nitrile imines with amino acid esters, followed by DDQ-mediated chemoselective oxidation of the corresponding 4,5-dihydro intermediates. The compounds were evaluated for antifungal, antibacterial, and cytotoxic activities. The synthesized derivatives displayed selective antifungal activity, whereas only weak antibacterial and cytotoxic effects were observed. The highest potency was observed for C(5)-unsubstituted derivatives, which exhibited the broadest antimicrobial spectrum and inhibited the growth of *Candida albicans*, *Trichophyton rubrum*, and *Epidermophyton floccosum* with MIC_50_ values below 4 mg/L. In addition, the dermatophytes showed a biphasic dose-dependent response, characterized by growth stimulation at low concentrations and inhibition at higher concentrations, consistent with hormesis. These findings identify trifluoromethylated 1,2,4-triazin-6(1*H*)-ones, particularly C(5)-unsubstituted derivatives, as promising scaffolds for the development of new antifungal agents.

## 1. Introduction

One of the major challenges facing modern society is to reduce the impact of civilization diseases, including obesity, diabetes, cardiovascular disorders, cancer, and infections caused by drug-resistant pathogens. Among these, fungal infections deserve particular attention, as more than 6.5 million people develop life-threatening fungal diseases each year [1]. Of all fungal species identified to date, only a relatively small fraction (approximately 300 species) is pathogenic to humans, with the majority belonging to the genera *Candida*, *Aspergillus*, *Cryptococcus*, and *Pneumocystis* [2]. In recent years, the incidence of fungal infections has increased steadily, largely due to the emergence of resistance to the relatively limited number of antifungal agents currently available [3]. Furthermore, this trend is expected to intensify as climate change and environmental adaptation enable previously non-pathogenic fungal species to tolerate higher temperatures and eventually acquire the ability to infect humans [4].

**Figure 1:**
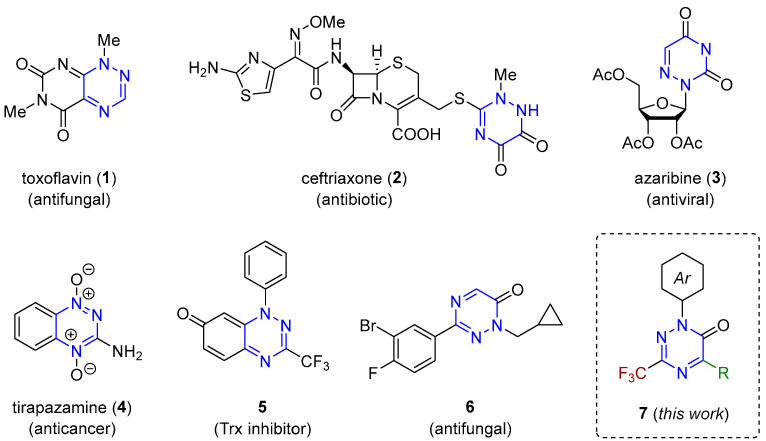
Structures of selected 1,2,4-triazine-based bioactive compounds **1**–**6** and the CF_3_-triazin-6-ones **7** reported herein.

Owing to their close structural similarity to the pyrimidine bases found in nucleic acids, 1,2,4-triazines and their analogues represent an important structural motif present in numerous natural products as well as synthetic pharmaceuticals [5,6]. A representative example is toxoflavin (**1**), a naturally occurring compound containing a 1,2,4-triazine ring that exhibits remarkable antifungal activity (Figure 1). Toxoflavin is produced by *Burkholderia gladioli*, originally isolated from *Lycoris aurea*, and displays fungicidal activity not only against plant pathogens but also against clinically relevant fungi, including *Candida albicans*, *Aspergillus fumigatus*, and *Cryptococcus neoformans*, even in azole-resistant strains [7]. A variety of synthetic 1,2,4-triazine derivatives have likewise been reported to exhibit antifungal activity against *C. albicans*, *A. fumigatus*, and *C. neoformans* [8]. More structurally complex benzo[4,5]imidazo[1,2-*d*][1,2,4]triazine derivatives have also emerged as a promising class of fungicides capable of suppressing the growth of phytopathogenic fungi responsible for substantial agricultural losses [9].

Notably, 1,2,4-triazine derivatives exhibit a broad spectrum of biological activities extending beyond antifungal properties to include antibacterial and antiviral effects, which have been successfully exploited in modern drug development. One of the best-known representatives of this class is ceftriaxone (**2**), a third-generation cephalosporin with potent activity against both Gram-positive and Gram-negative bacteria. Ceftriaxone is widely used in the treatment of severe bacterial infections such as typhoid fever, bacterial meningitis, and sepsis [6]. Another representative compound is azaribine (**3**), a prodrug possessing well-documented antiviral activity [10].

Numerous studies have also demonstrated that compounds incorporating the 1,2,4-triazine scaffold may effectively inhibit cancer cell proliferation, making them attractive candidates for the development of novel anticancer therapeutics. One notable example is tirapazamine (**4**), a bio-reductive drug that becomes selectively activated under hypoxic conditions characteristic of many solid tumors [11]. Upon activation, tirapazamine generates highly reactive radical species capable of inducing extensive DNA damage, ultimately leading to selective cancer cell death. Other derivatives containing the 1,2,4-triazin-6-one core have been shown to inhibit the migration of cervical cancer cells, thereby reducing their metastatic potential [12]. Moreover, several 1,2,4-triazinone derivatives exhibit promising antiproliferative activity against K-562 leukemia cells and MCF-7 breast cancer cells, as well as androgen receptor-dependent prostate cancer cells [13,14,15]. Despite the considerable pharmacological potential of this scaffold, fluorinated analogues—particularly trifluoromethyl-substituted derivatives—remain relatively unexplored. This is most likely attributable to the limited availability of suitable fluorinated synthetic precursors and the challenges associated with their preparation [16].

Collectively, these findings clearly demonstrate that the discovery of new bioactive compounds based on the 1,2,4-triazin-6-one scaffold remains an important research direction at the interface of organic chemistry, biology, and medicinal science. Particular attention has been devoted to compounds combining high biological activity with synthetic accessibility and the use of inexpensive, readily available starting materials. In this context, the present work aimed to develop an efficient synthetic methodology and to evaluate the anticancer, antibacterial, and antifungal activities of a new class of trifluoromethylated 1,2,4-triazine-based heterocycles.

## 2. Results

### 2.1. Design and Synthesis

The target compounds were designed on the basis of literature reports describing the promising biological activity of selected 1,2,4-triazine derivatives, particularly compound **5** (thioredoxin reductase inhibitor), which incorporates both a trifluoromethyl substituent and a redox-active quinone moiety (Figure 1) [17]. Additional inspiration was drawn from reports on halogenated 1,2,4-triazin-6-one derivatives such as **6** exhibiting significant activity against agriculturally relevant plant-pathogenic fungi [18]. In this context, the incorporation of a fluoroalkyl substituent in **7** was considered a key structural feature intended to increase molecular lipophilicity and, consequently, improve membrane permeability and potentially enhance pharmacokinetic properties [19]. Furthermore, additional potentially important pharmacophoric element i.e., the conjugated N=C–C=O system embedded in the triazine ring of **7**, was identified within the designed molecules.

Another major objective of this project was the development of an efficient and versatile synthetic route based on inexpensive and readily available starting materials. These requirements are fulfilled by commercially available α-amino acid esters as well as hydrazonoyl halides, which serve as convenient precursors of key 1,3-dipoles, namely fluorinated nitrile imines [20,21]. The reactivity of these intermediates has been the subject of a series of recent studies conducted by our group as well as by other research groups [22,23,24,25,26,27,28,29]. Accordingly, the proposed synthetic strategy exploits the 1,3-dipolar nature of nitrile imines, enabling their application not only in the well-established (3+2)-cycloaddition reactions with dipolarophiles [20,21,27,28,29], but also in the less explored (3+3)-annulation reactions with appropriately designed reagents containing geminal nucleophilic and electrophilic reaction centers [30,31,32]. This approach provides an efficient route toward the synthesis of novel fluorinated heterocyclic systems with expected biological potential.

**Scheme 1:**
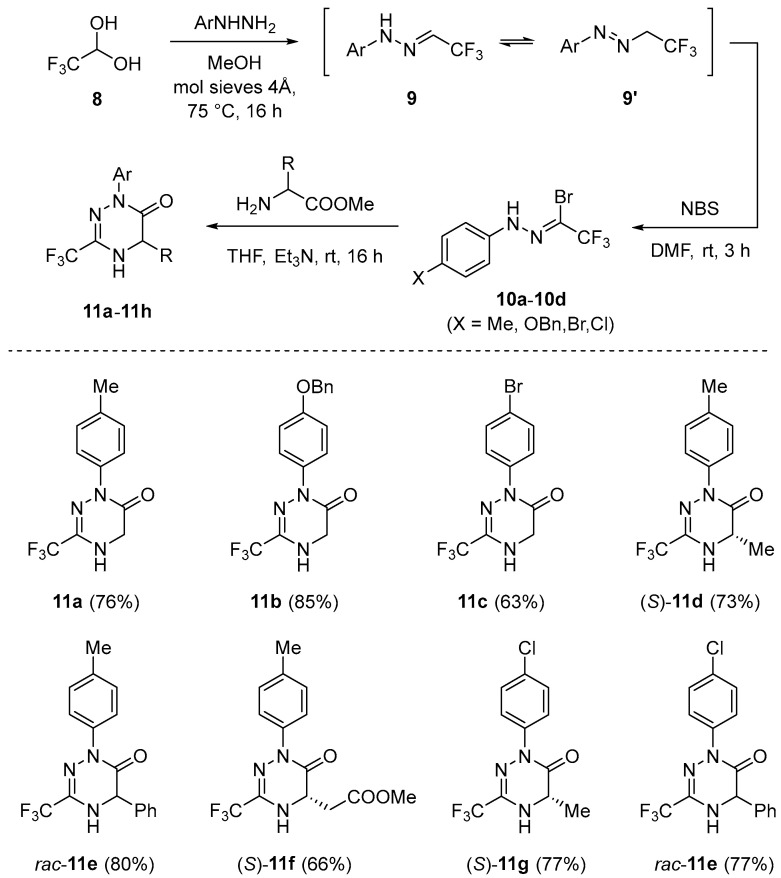
Synthesis of 4,5-dihydro-1,2,4-triazines **11a**–**11h** via trapping of methyl esters of amino acids by in situ-generated nitrile imines derived from hydrazonoyl bromides **10a**–**10d**.

As shown in Scheme 1, the first step of the designed synthetic sequence involved the thermal condensation (75 °C) of aqueous fluoral hydrate (**8**, ~75% in H_2_O) with commercial arylhydrazines. The reaction was carried out according to the reported procedure in the presence of freshly activated molecular sieves in a tightly sealed ampoule [33]. Subsequently, the crude trifluoroacetaldehyde arylhydrazones **9**, which exist in solution in equilibrium with their azo tautomer **9**′, were subjected to selective bromination at the azomethine carbon using *N*-bromosuccinimide (NBS) [22,34]. This protocol afforded a series of four known hydrazonoyl bromides **10a**–**10d** (X = Me, OBn, Br, and Cl), which served as precursors for the corresponding nitrile imines generated in situ via base-promoted dehydrobromination with triethylamine (Et_3_N).

The in situ-generated nitrile imines derived from bromides **10** underwent (3+3)-cycloaddition reactions with methyl esters of selected amino acids under mild conditions (THF, room temperature, overnight), furnishing the expected 4,5-dihydro-1,2,4-triazin-6-ones of type **11** [35]. The products were isolated by column chromatography in good to excellent yields (52–85%). In this manner, a series of eight compounds was prepared, including three achiral glycine derivatives (**11a**–**11c**; X = Me, OBn, Br), three enantiomerically pure derivatives obtained from l-alanine (**11d**,**g**; X = Me, Cl) and dimethyl l-aspartate (**11f**; X = Me), as well as two racemic products derived from racemic phenylglycine (**11e**,**h**; X = Me, Cl). In our previous work, the highly epimerization-prone N(4)-methylated phenylglycine-derived 4,5-dihydro-1,2,4-triazine was analyzed by HPLC using both enantiopure and racemic samples, demonstrating that no loss of optical purity occurred [35]. Based on this observation, we assumed that the analogous intermediates **11d**, **11f**, and **11g**, derived from optically pure amino acids were likewise obtained in enantiopure form.

**Scheme 2:**
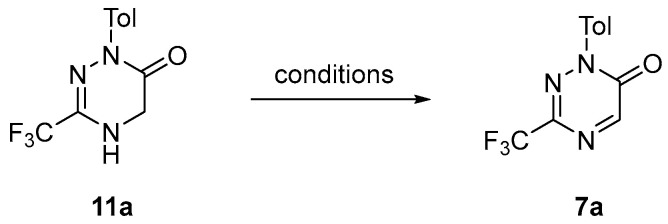
Oxidation of the N(4)–C(5) bond in 4,5-dihydro-1,2,4-triazin-6(1*H*)-one **11a**.

Studies on the chemoselective oxidation of the N(4)–C(5) bond in 4,5-dihydro-1,2,4-triazin-6(1*H*)-ones **11** commenced with the evaluation of suitable oxidizing agents. Initially, activated manganese(IV) oxide was examined using model substrate **11a** (Scheme 2, Table 1). However, regardless of the solvent employed (CH_2_Cl_2_ or THF) or the reaction temperature (room temperature or 60 °C), no formation of the desired oxidation product was observed. Subsequently, two additional readily available oxidants, namely 2,3-dichloro-5,6-dicyano-1,4-benzoquinone (DDQ) and potassium ferricyanide (K_3_[Fe(CN)_6_]), were investigated. Both reagents afforded the expected 1,2,4-triazin-6-one **7a** as the sole reaction product. In the case of DDQ, complete conversion was achieved after 24 h under reflux in ethyl acetate (77 °C), whereas oxidation with potassium ferricyanide required 48 h at room temperature to lead the reaction to completion. Compound **7a** was purified by column chromatography and isolated as a colorless solid in comparable yields (79% and 69%, respectively), and its structure was confirmed by NMR spectroscopy.

**Figure 2:**
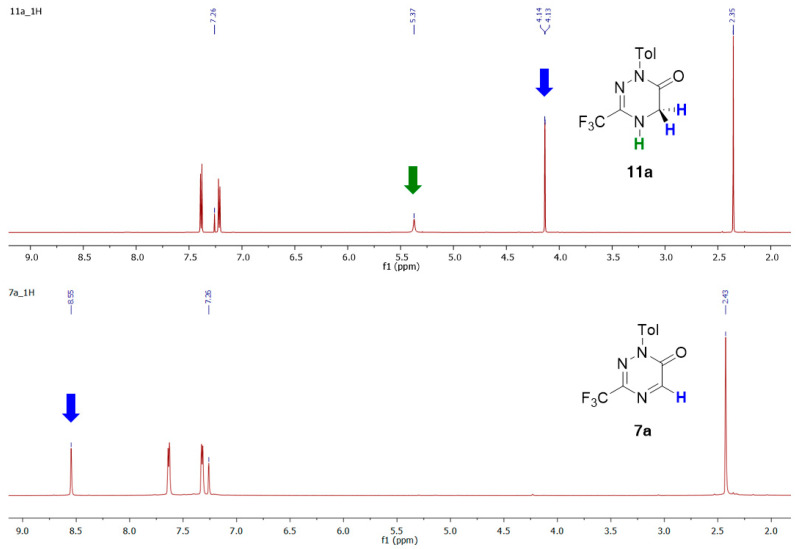
Diagnostic chemical shifts in ^1^H NMR (600 MHz, CDCl_3_) spectra of **11a** and **7a**.

As shown in Figure 2, the ^1^H NMR spectrum of substrate **11a** displayed the characteristic signal pattern of the –CH_2_NH– unit, including a broad singlet at δ = 5.37 assigned to the NH proton and a broad doublet at δ = 4.14 (*J* ≈ 1.5 Hz) corresponding to the two methylene protons located at C(5). As expected, the ^1^H NMR spectrum of the oxidized product **7a** exhibited, in addition to the signals of the *p*-tolyl substituent, a characteristic downfield singlet at δ = 8.55 assigned to the proton attached to the sp^2^-hybridized C(5) atom, thereby unequivocally confirming the selective oxidation of the N(4)–C(5) bond.

**Figure 3:**
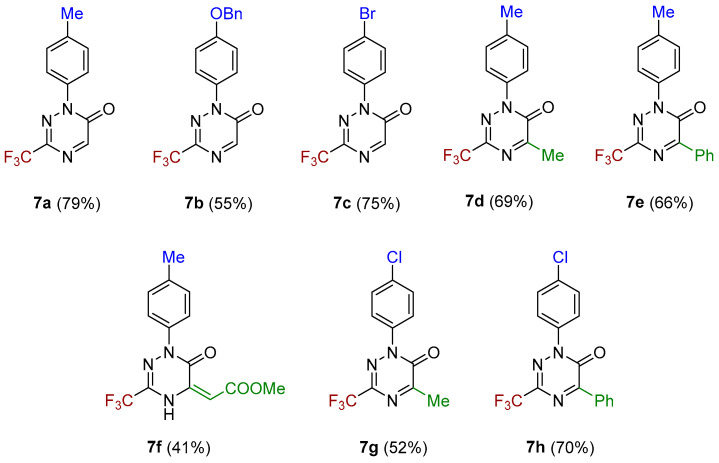
Structures of target 3-trifluoromethyl-1,2,4-triazin-6-ones **7a**–**7h**.

Since subsequent studies revealed that potassium ferricyanide provided inferior results for the oxidation of other 4,5-dihydro-1,2,4-triazine derivatives (**11b**, **11c**, and **11e**), the entire series of target compounds **7a**–**7h** was ultimately synthesized using DDQ in refluxing ethyl acetate (Figure 3).

The investigated 4,5-dihydro-1,2,4-triazine derivatives **11** proved to be highly susceptible to chemoselective oxidation with DDQ, affording the corresponding 1,2,4-triazines **7** in generally good yields. However, in the case of compounds **7b** and **7g**, partial decomposition was observed during purification by column chromatography, resulting in diminished isolated yields of 55% and 52%, respectively. It is also worth noting that oxidation of the aspartic acid-derived 4,5-dihydro-1,2,4-triazine **11f** afforded a different product containing an exocyclic alkylidene double bond (Figure 3). This compound is presumed to arise via a 1,3-prototropic shift of the initially formed 1,2,4-triazine-6(1*H*)-one, leading to the thermodynamically more stable conjugated tautomer **7f**. The structures of all products **7a**–**7h** were confirmed on the basis of ^1^H, ^13^C, and ^19^F NMR spectroscopy, supplemented by IR measurements and HMRS or combustion analysis (Supporting Information, Figures S1–S17). Thus, in addition to resonances of the N(1)-aryl substituents, the ^1^H NMR spectra of compounds **7a**–**7h** exhibited highly characteristic singlets corresponding to the C(5)-H proton in **7a**–**7c** at δ = 8.53–8.56. In contrast, the expected singlet of the C(5) methyl group at δ ≈ 2.67 in **7d** and **7g**, as well as the characteristic aromatic resonances of the phenyl group in **7e** and **7h**, confirmed substitution at the C(5) position of the 1,2,4-triazine ring. Furthermore, the ^19^F NMR spectra of all compounds displayed a singlet at δ ≈ −69.8, confirming the presence of the CF_3_ group. In the ^13^C NMR spectra of **1a**–**1e**, **1g**, and **1h**, characteristic quartets assigned to the CF_3_ carbon and C(3) were observed at δ = 119.1–119.5 (^1^*J*_C-F_ = 273.4–273.9 Hz) and δ = 139.8–140.0 (^2^*J*_C-F_ = 38.1–39.3 Hz), respectively. Only the methylidene derivative **7f** showed significant up-field shifts of these signals to δ = 117.9 (^1^*J*_C-F_ = 274.7 Hz) and δ = 131.3 (^2^*J*_C-F_ = 39.6 Hz), respectively. The carbonyl group of the 1,2,4-triazin-6-one ring was identified by signals at δ = 152.3–153.2 in the ^13^C NMR spectra and by strong IR absorption at 1633–1688 cm^−1^. In addition, compound **7f** exhibited an additional carbonyl resonance at δ = 170.4 and an IR absorption at 1692 cm^−1^, consistent with a conjugated ester functionality. Finally, ESI mass spectrometry confirmed the molecular formulas of all compounds **7a**–**7h**.

### 2.2. Evaluation of Biological Activity

#### 2.2.1. Screening of Antifungal Properties

**Figure 4:**
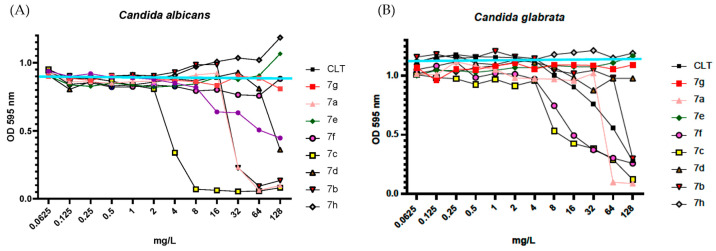
Antifungal activity of 1,2,4-triazines **7a**–**7h**: (**A**) *Candida albicans* and (**B**) *Candida glabrata*. The blue line indicates the optical density (OD_595_) of the untreated control culture. CLT—clotrimazole, used as a reference drug.

The antifungal activity of the target 1,2,4-triazin-6(1*H*)one derivatives (**7a**–**7h**) was evaluated over a concentration range of 0.0625–128 mg/L against *Candida albicans*, *Candida glabrata*, *Trichophyton rubrum*, and *Epidermophyton floccosum*. Following 48 h of incubation of *C. albicans* and *C. glabrata*, fungal growth was quantified by measuring the optical density at 595 nm (OD_595_). The optical density values as a function of compound concentration are presented in Figure 4.

As shown in Figure 4, out of eight tested compounds, four derivatives **7a**–**7d** exhibited antifungal activity against *C. albicans*. Compound **7d** inhibited fungal growth only at the highest tested concentration (128 mg/L), whereas compounds **7a** and **7b** displayed comparable activity, inhibiting fungal growth at 32 mg/L. Among all derivatives, the 4-bromophenyl-substituted derivative **7c** proved to be the most potent. At 4 mg/L, it caused a pronounced reduction in the optical density (OD < 0.5) relative to the untreated control, while nearly complete growth inhibition was achieved at 8 mg/L.

Against *C. glabrata*, 1,2,4-triazines **7c** and **7f** displayed comparable antifungal activity; however, **7c** was clearly more potent, reducing the optical density below 0.5 at 8 mg/L, whereas the 5-alkylidene derivative **7f** produced a similar effect only at 16 mg/L. The 5-unsubstituted 1,2,4-triazine **7a**, bearing a *p*-tolyl substituent at the N(1) position, inhibited fungal growth only at the two highest tested concentrations (64 and 128 mg/L), while the corresponding 4-benzyloxyphenyl analogue **7b** was active exclusively at 128 mg/L.

Notably, the compounds active against *C. albicans* reduced fungal growth more efficiently than the reference antifungal agent clotrimazole. Whereas all active compounds decreased the optical density below 0.5, the lowest OD value recorded for clotrimazole at 128 mg/L was approximately 0.5. A similar trend was observed for *C. glabrata*, although compounds **7b** and **7f** produced OD values comparable to or slightly higher than that of clotrimazole at the highest concentration. For clotrimazole, the lowest OD value against *C. glabrata* at 128 mg/L was approximately 0.3.

**Figure 5:**
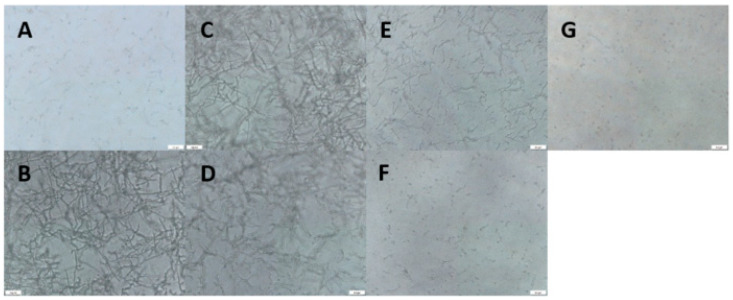
Microscopic analysis of the growth of germinating conidia of *Trichophyton rubrum* in the presence of increasing concentrations of 1,2,4-triazine **7a**. Image (**A**) shows the untreated control (*T. rubrum* cultured in the absence of the tested compound), whereas images (**B**–**G**) correspond to increasing concentrations of the compound: 0.0625, 0.25, 1, 4, 16, and 32 mg/L, respectively. Magnification: ×400. The white scale bar represents 100 µm.

The antifungal activity of 1,2,4-triazines **7a**–**7h** against *Trichophyton rubrum* and *Epidermophyton floccosum* was assessed by microscopic examination of germinating conidia, as extensive mycelial growth precluded reliable spectrophotometric determination of optical density. Representative microscopic images illustrating the effect of compound **7a** on the growth of *T. rubrum* are shown in Figure 5, while additional images are provided in the Supporting Information (Figures S18–S32 and Tables S1 and S2). Interestingly, at low concentrations of the tested compounds, both dermatophyte species exhibited more vigorous mycelial growth than the untreated controls.

The collected results demonstrated that 1,2,4-triazin-6(1*H*)-ones **7a** and **7g** exhibited comparable antifungal activity against *T. rubrum*, inhibiting mycelial development at 16 mg/L. Although the MIC_50_ values of the more sterically demanding diaryl-substituted derivatives **7e** and **7h** could not be determined within the investigated concentration range, a gradual concentration-dependent reduction in mycelial growth was observed. Compounds **7d** and **7f** inhibited fungal growth at 32 mg/L, whereas compound **7b** exhibited relatively weak activity, with growth inhibition occuring only at 64 mg/L. As observed for the *Candida* species, the 4-bromophenyl derivative **7c** proved to be the most active compound with an MIC_50_ of 4 mg/L.

The activity profile against *E. floccosum* differed slightly from that observed for *T. rubrum*. At the lowest tested concentration (0.0625 mg/L), only limited mycelial growth was observed for all examined compounds of type **7**. As the concentration increased, mycelial growth initially became more extensive before gradually declining at higher concentrations, eventually leading to growth inhibition. Compounds **7a**, **7d**, and **7g** exhibited MIC_50_ values of 4 mg/L, whereas compound **7f** inhibited fungal growth at 16 mg/L. The diaryl-substituted derivative **7e** showed the weakest antifungal activity, with an MIC_50_ of 64 mg/L, while its structural analogue **7h** displayed only modest activity, with growth inhibition first observed at 16 mg/L. In contrast, the C(5)-unsubstituted derivatives **7b** and **7c** were the most potent compounds in this series, exhibiting MIC_50_ values of 1 mg/L.

#### 2.2.2. Analysis of Antimicrobial Activity

**Figure 6:**
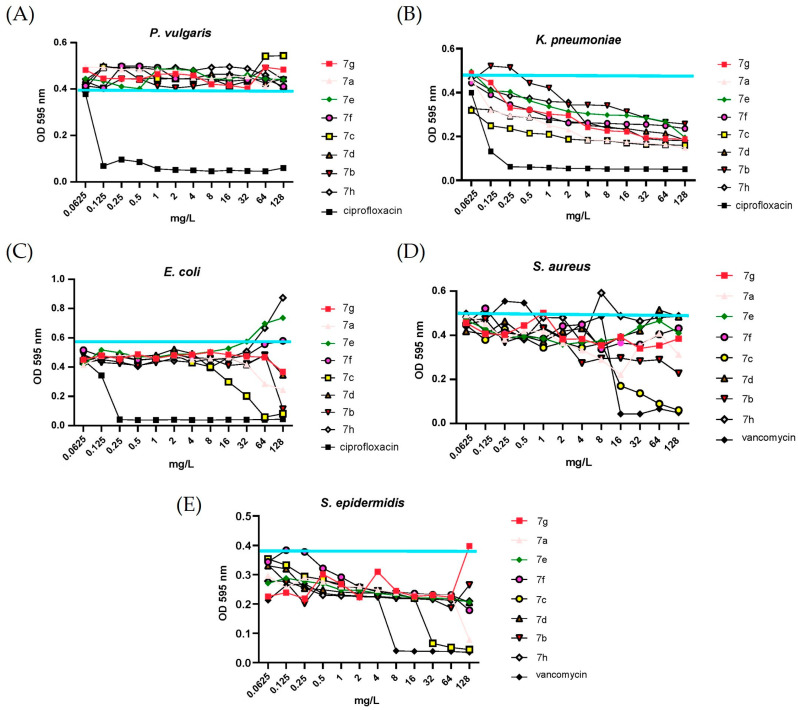
Antibacterial activity of 1,2,4-triazines **7a**–**7h** against the reference bacterial strains: (**A**) *Proteus vulgaris*, (**B**) *Klebsiella pneumoniae*, (**C**) *Escherichia coli*, (**D**) *Staphylococcus aureus*, and (**E**) *Staphylococcus epidermidis*. The blue line indicates the optical density (OD) of the untreated control culture. Ciprofloxacin was used as the reference drug for *P. vulgaris*, *K. pneumoniae*, and *E. coli*, while vancomycin was used for *S. aureus* and *S. epidermidis*.

The antibacterial activity of 1,2,4-triazine derivatives **7a**–**7h** was evaluated against the reference strains of the Gram-positive bacteria *Staphylococcus aureus* and *Staphylococcus epidermidis*, as well as the Gram-negative bacteria *Proteus vulgaris*, *Klebsiella pneumoniae*, and *Escherichia coli*, over a concentration range of 0.0625–128 mg/L. After 24 h of incubation, bacterial growth was assessed by measuring the optical density at 595 nm (OD_595_). The collected results are presented in Figure 6.

None of the investigated compounds exhibited antibacterial activity against *P. vulgaris*. Derivatives **7a**, **7c**, **7d**, **7e**, **7g**, and **7h** displayed weak antibacterial activity against *K. pneumoniae*, with MIC_50_ values of 4, 2, 128, 128, 32, and 128 mg/L, respectively. Additionally, compound **7a** showed weak activity against *E. coli* (MIC_50_ = 64 mg/L) and inhibited the growth of *S. epidermidis* only at the highest tested concentration (128 mg/L). Similarly, compound **7b** inhibited the growth of *E. coli* at 128 mg/L and exhibited weak activity against *S. aureus* at the same concentration.

Among all the investigated derivatives, compound **7c** demonstrated the broadest and most potent antibacterial activity. It inhibited the growth of *S. aureus*, *S. epidermidis*, and *E. coli* with MIC_50_ values of 16, 32, and 64 mg/L, respectively. When compared with the reference antibiotics, the 4-bromophenyl analogue **7c** was the only derivative that inhibited bacterial growth with comparable efficacy, although only at concentrations of 64 mg/L or higher.

#### 2.2.3. Cytotoxicity Studies

All 1,2,4-triazine derivatives of type **7** were evaluated for cytotoxic activity against four selected human cancer cell lines, namely promyelocytic leukemia (HL-60), breast adenocarcinoma (MCF-7), lung carcinoma (A549), and prostate carcinoma (DU-145), as well as normal, non-cancerous human umbilical vein endothelial cells (HUVECs), using the MTT assay over a concentration range of 1–200 μM (Table 2). Among the eight compounds tested, three derivatives (**7a**–**7c**) lacking a substituent at the C5 position of the 1,2,4-triazine ring exhibited promising cytotoxic activity against HL-60 cells, with IC_50_ below 10 μM, and moderate activity against the remaining cancer cell lines. However, these derivatives were essentially nonselective, displaying comparable cytotoxicity toward HL-60 cells and normal HUVECs and even greater toxicity toward HUVECs than toward the MCF-7, A549, and DU-145 cancer cell lines. Only compound **7a** exhibited modest selectivity, being approximately twofold less cytotoxic toward HUVECs than toward HL-60 cells.

Notably, the introduction of such substituents as small alkyl (Me in **7d**,**g**), aryl (Ph in **7e**,**h**), or alkylidene functional group =CCO_2_Me (**7f**) at the C(5) position of the heterocyclic ring resulted in a complete loss of cytostatic activity.

## 3. Discussion

A series of 1,2,4-triazine derivatives **7a**–**7h** bearing a CF_3_ group at C(3) and an oxo functionality at C(6) was designed based on recent reports describing the biological activity of molecular systems incorporating the 1,2,4-triazine scaffold [5,6,7,8,9,10,11,12,13,14,15]. The key heterocyclic core was accessed through a known strategy involving a (3+3)-cycloaddition of in situ-generated CF_3_-nitrile imines with amino acid esters [35]. Subsequent oxidation studies identified DDQ as a suitable reagent for the fully chemoselective oxidation of the N(4)–C(5) bond, providing the desired products in fair overall yields of 27–60%. The substitution pattern on the final materials was dictated by the structure of the starting materials; in particular the amino acid ester determined the substituent located at C(5) (H, Me, Ph, or =CHCO_2_Me). In addition, *para*-substituted phenyl groups bearing substituents ranging from strongly electron-donating (OBn), through weakly electron-donating (Me), to moderately electron-withdrawing (halogens; Cl or Br) were introduced at the N(1) position of the 1,2,4-triazine ring. The resulting series of eight model compounds was evaluated for antimicrobial and anticancer activities.

The biological evaluation of the target compounds included the assessment of their antimicrobial activity against representative fungal species (*Candida albicans*, *Candida glabrata*, *Trichophyton rubrum*, and *Epidermophyton floccosum*) and bacterial strains (*Staphylococcus aureus*, *Staphylococcus epidermidis*, *Klebsiella pneumoniae*, *Escherichia coli*, and *Proteus vulgaris*), as well as the determination of their cytotoxicity toward selected human cancer cell lines (HL-60, MCF-7, A549, and DU-145) and normal human endothelial cells (HUVECs). Overall, the synthesized compounds of type **7** displayed an interesting selectivity profile, exhibiting promising antifungal activity accompanied by only weak antibacterial and cytotoxic effects.

In general, the highest activity was observed against *Candida* species, with compounds **7a**–**7c**, lacking a substituent at the C(5) position of the 1,2,4-triazine ring, showing the most pronounced antifungal properties. Against *C. albicans*, compounds **7a**–**7c**, and C(5)-methylated product **7d** inhibited fungal growth with MIC_50_ values of 32, 32, 4, and 128 mg/L, respectively. For *C. glabrata*, MIC_50_ values of 64, 128, 16, and 16 mg/L were determined for compounds **7a**–**7c** and C(5)-alkylidene-functionalized analogue **7f**, respectively. Notably, for most of the active compounds, fungal growth inhibition at the corresponding MIC_50_ values was more pronounced than that observed for the reference antifungal agent clotrimazole.

Notably, an unusual dose-dependent response was observed for the dermatophytes. Whereas high concentrations of the tested compounds markedly inhibited fungal growth, lower concentrations promoted fungal proliferation. Such biphasic, concentration-dependent responses are characteristic of hormesis [36], a phenomenon that has only rarely been reported for fungi [37,38]. For example, hormesis has previously been described for an *N*-(3,5-dichlorophenyl)succinimide, a well-known fungicide used against *Sclerotinia sclerotiorum*, recognized as widespread phytopathogenic fungus infecting numerous crop species [39]. Interestingly, the hormesis response was observed irrespective of the resistance phenotype, occurring in both fungicide-sensitive and fungicide-resistant strains.

In contrast to their antifungal properties, all synthesized derivatives **7a**–**7h** displayed only weak antibacterial activity. Overall, the compounds were slightly more active against Gram-positive than Gram-negative bacteria, which may be attributed to the structural differences in the bacterial cell envelope, particularly the presence of the outer membrane in Gram-negative bacteria that limits the penetration of xenobiotics into the bacterial cell. Among the investigated compounds, the C(5)-unsubstituted derivatives **7a**–**7c** inhibited the growth of *K. pneumoniae*, *E. coli*, *S. aureus*, and *S. epidermidis*, whereas compounds **7d**, **7e**, **7g**, and **7h** were active against *K. pneumoniae* exclusively, and only at the highest concentrations tested. In contrast, *P. vulgaris* was completely insensitive to all evaluated compounds.

Among the synthesized derivatives, compound **7c** bearing a *para*-bromophenyl substituent exhibited the most favorable biological profile and the broadest antimicrobial spectrum, inhibiting the growth of yeasts, dermatophytes, Gram-positive bacteria, and Gram-negative bacteria, with the exception of *P. vulgaris*. This finding is consistent with previous reports demonstrating that the introduction of halogenated aryl substituents into heterocyclic scaffolds frequently enhances biological activity [40,41]. For example, similar beneficial effects have been reported for thiazole-based antifungal agents active against *Cryptococcus neoformans*, *Aspergillus fumigatus*, and *Candida albicans* [42].

The cytotoxicity studies revealed only weak antiproliferative activity, which was limited to compounds **7a**–**7c**. However, these derivatives also exhibited measurable toxicity toward normal HUVEC cells, with IC_50_ values of 23.04 ± 1.72 μM (ca. 5.9 mg/L), 3.99 ± 0.15 μM (ca. 1.4 mg/L), and 5.86 ± 0.14 μM (ca. 1.9 mg/L), respectively. These findings indicate that the concentrations required to effectively inhibit *Candida* growth may also induce cytotoxic effects in normal human cells, thereby limiting the therapeutic potential of the most active 1,2,4-triazine derivatives as antifungal agents. Nevertheless, the observed structure–activity relationships suggest that further structural optimization of the title trifluoromethylated 1,2,4-triazin-6(1*H*)-ones is warranted in order to enhance their antifungal potency while simultaneously reducing their cytotoxicity. Furthermore, the remarkable activity of C(5)-unsubstituted analogues **7a**–**7c** in comparison to structural analogues **7d**–**7h** may indicate a crucial role of the imine moiety (CH=NH) in the biological activity of these compounds, possibly through the generation of a corresponding aminyl radicals [43,44]. Therefore, further efforts to identify cytotoxic agents within the investigated series of 3-(trifluoromethyl)-1,2,4-triazin-6(1*H*)-ones should focus on structural modifications of the substituent at the remaining positions.

## 4. Materials and Methods

### 4.1. Chemical Synthesis—General Methods

Experimental procedures: If not stated otherwise, all organic solvents and chemicals were purchased and used as received. Products were purified by standard column chromatography (CC) on silica gel (230–400 mesh). Melting points were determined in capillaries using a MEL-TEMP II apparatus (Laboratory Devices, Holliston, MA, USA) or a Stuart SMP30 apparatus (Bibby Scientific Ltd., Stone, Staffordshire, UK) with automatic temperature monitoring and are uncorrected. NMR spectra were recorded on a Bruker AVIII instrument (^1^H at 600 MHz, ^13^C at 151 MHz, and ^19^F at 565 MHz) (Bruker BioSpin AG, Fällanden, Switzerland). Chemical shifts are reported relative to residual solvent signals (CDCl_3_: ^1^H NMR: δ = 7.26, ^13^C NMR: δ = 77.16; CD_3_OD: ^1^H NMR: δ = 3.31) or relative to CFCl_3_ (^19^F NMR: δ = 0.00) used as an external standard. IR spectra were measured neat with an Agilent Cary 630 FTIR spectrometer (Agilent Technologies, Santa Clara, CA, USA). Mass spectra (ESI) were acquired using a Varian 500-MS LC Ion Trap instrument (Varian, Inc., Walnut Creek, CA, USA), and high resolution MS (ESI-TOF) measurements were taken with a Waters Synapt G2-Si mass spectrometer (Waters Corporation, Milford, MA, USA). Optical rotations were measured using an MCP 500 polarimeter (Anton Paar, Graz, Austria) at the temperatures indicated. Combustion analyses were performed using a Vario EL III instrument (Elementar Analysensysteme GmbH, Langenselbold, Germany).

#### 4.1.1. General Procedure for Synthesis of 1,2,4-Triazin-6(1*H*)-ones **7a**–**7h**

To a solution of 4,5-dihydro-1,2,4-triazin-6(1*H*)-one **11** (1.0 mmol) in EtOAc (10 mL) was added DDQ (341 mg, 1.5 mmol). The resulting reaction mixture was refluxed for 24 h. After cooling to room temperature, the solvent was removed under reduced pressure. The residue was dissolved in dichloromethane (50 mL) and washed with saturated aqueous Na_2_CO_3_ solution (3 × 20 mL). The organic layer was dried over anhydrous MgSO_4_, filtered, and concentrated under reduced pressure. The crude product **7** was purified by standard column chromatography or recrystallization.

1-(*p*-Tolyl)-3-(trifluoromethyl)-1,2,4-triazin-6(1*H*)-one (**7a**): CC (SiO_2_, DCM); colorless solid, 202 mg (79%), mp = 80–82 °C. ^1^H NMR (600 MHz, CDCl_3_) δ 2.43 (s, 3H), 7.31–7.34 (m, 2H), 7.62–7.65 (m, 2H), 8.55 (s, 1H). ^13^C NMR (151 MHz, CDCl_3_) δ 21.4, 119.2 (q, ^1^*J*_C-F_ = 273.4 Hz, CF_3_), 123.9, 129.9, 136.6, 140.2, 140.7 (q, ^2^*J*_C-F_ = 38.9 Hz, C(3)), 152.7 (C=O), 161.1. ^19^F NMR (565 MHz, CDCl_3_) δ −69.67 (s, CF_3_). IR (neat) *ν* 1674 (C=O), 1599, 1510, 1394, 1346, 1208, 1156, 1088 cm^−1^. ESI-MS (*m*/*z*): 256.2 (100, [M+H]^+^). Anal. Calcd (%) for C_11_H_8_F_3_N_3_O (255.2): C 51.77, H 3.16, N 16.47; found: C 51.61, H 3.16, N 16.51.

1-(4-Benzyloxyphenyl)-3-(trifluoromethyl)-1,2,4-triazin-6(1*H*)-one (**7b**): CC (SiO_2_, DCM); yellow solid, 191 mg (55%), mp = 89–91 °C. ^1^H NMR (600 MHz, CDCl_3_) δ 5.13 (s, 2H), 7.07–7.10 (m, 2H), 7.33–7.36 (m, 1H), 7.39–7.44 (m, 4H), 7.69–7.72 (m, 2H), 8.53 (s, 1H). ^13^C NMR (151 MHz, CDCl_3_) δ 70.6, 115.4, 119.2 (q, ^1^*J*_C-F_ = 273.6 Hz, CF_3_), 125.5, 127.6, 128.4, 128.9, 132.3, 136.4, 140.7 (q, ^2^*J*_C-F_ = 39.0 Hz, C(3)), 152.7 (C=O), 159.7, 160.9. ^19^F NMR (565 MHz, CDCl_3_) δ −69.64 (s, CF_3_). IR (neat) *ν* 1677 (C=O), 1510, 1390, 1252, 1193, 1141, 1088, 1021 cm^−1^. HRMS ((+)-ESI-TOF) *m*/*z*: [M+H]+ calcd for C_17_H_13_F_3_N_3_O_2_: 348.0960; found: 348.0958.

1-(4-Bromophenyl)-3-(trifluoromethyl)-1,2,4-triazin-6(1*H*)-one (**7c**): CC (SiO_2_, DCM); yellow solid, 241 mg (75%), mp = 99–100 °C. ^1^H NMR (600 MHz, CDCl_3_) δ 7.64–7.68 (m, 2H), 7.69–7.72 (m, 2H), 8.56 (s, 1H). ^13^C NMR (151 MHz, CDCl_3_) δ 119.1 (q, ^1^*J*_C-F_ = 273.8 Hz, CF_3_), 123.9, 125.5, 132.5, 138.0, 140.9 (q, ^2^*J*_C-F_ = 39.3 Hz, C(3)), 152.3 (C=O), 161.5. ^19^F NMR (565 MHz, CDCl_3_) δ −69.78 (s, CF_3_). IR (neat) *ν* 1688 (C=O), 1484, 1390, 1193, 1137, 1088 cm^−1^. HRMS ((+)-ESI-TOF) *m*/*z*: [M{^79^Br}+H]+ calcd for C_10_H_6_F_3_N_3_OBr: 319.9646; found: 319.9651.

5-Methyl-1-(*p*-tolyl)-3-(trifluoromethyl)-1,2,4-triazin-6(1*H*)-one (**7d**): CC (SiO_2_, DCM); pale yellow solid, 186 mg (69%), mp = 54–55 °C. ^1^H NMR (600 MHz, CDCl_3_) δ 2.42 (s, 3H), 2.67 (s, 3H), 7.30–7.31 (m, 2H), 7.59–7.61 (m, 2H). ^13^C NMR (151 MHz, CDCl_3_) δ 21.2, 21.4, 119.3 (q, ^1^*J*_C-F_ = 273.8 Hz, CF_3_), 124.2, 129.8, 137.1, 139.77, 139.79 (q, ^2^*J*_C-F_ = 38.3 Hz, C(3)), 153.5 (C=O), 172.5. ^19^F NMR (565 MHz, CDCl_3_) δ −69.95 (s, CF_3_). IR (neat) *ν* 1677 (C=O), 1607, 1506, 1394, 1234, 1204, 1118 cm^−1^. HRMS ((+)-ESI-TOF) *m*/*z*: [M+H]+ calcd for C_12_H_11_F_3_N_3_O: 270.0854; found: 270.0858.

5-Phenyl-1-(*p*-tolyl)-3-(trifluoromethyl)-1,2,4-triazin-6(1*H*)-one (**7e**): CC (SiO_2_, DCM); yellow solid, 218 mg (66%), mp = 126–127 °C. ^1^H NMR (600 MHz, CDCl_3_) δ 2.44 (s, 3H), 7.32–7.35 (m, 2H), 7.51–7.54 (m, 2H), 7.60–7.64 (m, 3H), 8.65–8.67 (m, 2H). ^13^C NMR (151 MHz, CDCl_3_) δ 21.4, 119.5 (q, ^1^*J*_C-F_ = 273.8 Hz, CF_3_), 124.7, 128.7, 129.8, 130.9, 132.9, 133.8, 137.5, 139.9, 140.6 (q, ^2^*J*_C-F_ = 38.1 Hz, C(3)), 153.2 (C=O), 163.1. ^19^F NMR (565 MHz, CDCl_3_) δ −69.73 (s, CF_3_). IR (neat) *ν* 1680 (C=O), 1506, 1453, 1394, 1274, 1189, 1122, 1052 cm^−1^. ESI-MS (*m*/*z*): 332.3 (100, [M+H]^+^). Anal. Calcd (%) for C_17_H_12_F_3_N_3_O (331.3): C 61.63, H 3.65, N 12.68; found: C 61.53, H 3.56, N 12.59.

Methyl[6-oxo-1-(*p*-tolyl)-3-(trifluoromethyl)-1,6-dihydro-1,2,4-triazin-5(4*H*)-ylidene]acetate (**7f**): CC (SiO_2_, DCM); pale yellow solid, 134 mg (41%), mp = 101–103 °C. ^1^H NMR (600 MHz, CDCl_3_) δ 2.39 (s, 3H), 3.82 (s, 3H), 6.11 (s, 1H), 7.24–7.27 (m, 2H), 7.45–7.48 (m, 2H), 11.69 (s_br_, 1H). ^13^C NMR (151 MHz, CDCl_3_) δ 21.3, 52.1, 92.4, 117.9 (q, ^1^*J*_C-F_ = 274.7 Hz, CF_3_), 124.6, 129.6, 131.3 (q, ^2^*J*_C-F_ = 39.6 Hz), 137.4, 138.5, 140.3, 152.9 (C=O), 170.4 (C=O). ^19^F NMR (565 MHz, CDCl_3_) δ −69.96 (s, CF_3_). IR (neat) *ν* 1692 (C=O), 1633 (C=O), 1446, 1390, 1338, 1297, 1245, 1156, 1118, 1081 cm^−1^. HRMS ((+)-ESI-TOF) *m*/*z*: [M+H]+ calcd for C_14_H_13_F_3_N_3_O_3_: 328.0909; found: 328.0908.

1-(4-Chlorophenyl)-5-methyl-3-(trifluoromethyl)-1,2,4-triazin-6(1*H*)-one (**7g**): CC (SiO_2_, DCM); pale yellow solid, 151 mg (52%), mp = 72–73 °C. ^1^H NMR (600 MHz, CDCl_3_) δ 2.67 (s, 3H), 7.46–7.48 (m, 2H), 7.71–7.73 (m, 2H). ^13^C NMR (151 MHz, CDCl_3_) δ 21.2, 119.2 (q, ^1^*J*_C-F_ = 273.8 Hz, CF_3_), 125.6, 129.4, 135.3, 137.8, 140.0 (q, ^2^*J*_C-F_ = 38.4 Hz, C(3)), 153.2 (C=O), 172.9. ^19^F NMR (565 MHz, CDCl_3_) δ −70.06 (s, CF_3_). IR (neat) *ν* 1681 (C=O), 1584, 1484, 1394, 1275, 1129, 1088 cm^−1^. HRMS ((−)-ESI-TOF) *m*/*z*: [M−H]^−^ calcd for C_11_H_6_F_3_N_3_OCl: 288.0151; found: 288.0160.

1-(4-Chlorophenyl)-5-phenyl-3-(trifluoromethyl)-1,2,4-triazin-6(1*H*)-one (**7h**): crude product was recrystallized from hexanes; pale yellow solid, 246 mg (70%), mp = 130–131 °C. ^1^H NMR (600 MHz, CDCl_3_) δ 7.49–7.54 (m, 4H), 7.63–7.65 (m, 1H), 7.72–7.75 (m, 1H), 8.63–8.65 (m, 2H). ^13^C NMR (151 MHz, CDCl_3_) δ 119.3 (q, ^1^*J*_C-F_ = 273.9 Hz, CF_3_), 126.1, 128.8, 129.4, 130.9, 132.7, 134.1, 135.5, 138.3, 140.9 (q, ^2^*J*_C-F_ = 38.5 Hz, C(3)), 153.0 (C=O), 163.4. ^19^F NMR (565 MHz, CDCl_3_) δ −69.82 (s, CF_3_). IR (neat) *ν* 1674 (C=O), 1554, 1487, 1442, 1398, 1279, 1193, 1122, 1081 cm^−1^. HRMS (APCI-TOF) *m*/*z*: [M]^+^ calcd for C_16_H_9_ClF_3_N_3_O: 351.0386; found: 351.0392.

#### 4.1.2. General Procedure for Synthesis of 4,5-Dihydro-1,2,4-triazin-6(1*H*)-ones **11a**–**11h**

To a suspension of the selected amino ester hydrochloride (2.0 mmol) in anhydrous THF (5 mL) under an inert argon atmosphere was added an excess of triethylamine (16.0 mmol, 2.24 mL). A solution of the hydrazonoyl bromide **10** (2.0 mmol) in dry THF (5 mL) was then added dropwise, and the reaction mixture was stirred overnight. After this time, the mixture was filtered, and the precipitate was washed with diethyl ether (2 × 40 mL). The combined filtrates were concentrated under reduced pressure to remove the solvents, and the crude product **11** was purified by standard column chromatography.

1-(*p*-Tolyl)-3-(trifluoromethyl)-4,5-dihydro-1,2,4-triazin-6(1*H*)-one (**11a**): CC (SiO_2_, 4% MeOH in DCM); colorless crystals, 390 mg (76%), mp = 149–151 °C. ^1^H NMR (600 MHz, CDCl_3_) δ 2.36 (s, 3H), 4.13 (d_br_, *J* ≈ 1.6 Hz, 2H), 5.40 (s_br_, 1H), 7.19–7.22 (m, 2H), 7.39–7.41 (m, 2H). ^19^F NMR (565 MHz, CDCl_3_) δ −70.58 (s, CF_3_). NMR data are in accordance with the literature [35].

1-(4-Benzyloxyphenyl)-3-(trifluoromethyl)-4,5-dihydro-1,2,4-triazin-6(1*H*)-one (**11b**): CC (SiO_2_, 2% MeOH in DCM); pale orange crystals, 592 mg (85%), mp = 129–131 °C. ^1^H NMR (600 MHz, CDCl_3_) δ 4.15 (d_br_, *J* ≈ 1.6 Hz, 2H), 5.07 (s, 2H), 5.37 (s_br_, 1H), 6.99–7.01 (m, 2H), 7.32–7.34 (m, 1H), 7.37–7.44 (m, 6H). ^19^F NMR (565 MHz, CDCl_3_) δ −70.51 (s, CF_3_). NMR data are in accordance with the literature [35].

1-(4-Bromophenyl)-3-(trifluoromethyl)-4,5-dihydro-1,2,4-triazin-6(1*H*)-one (**11c**): CC (SiO_2_, DCM gradient DCM/EtOAc 4:1); colorless solid, 406 mg (63%), mp = 130–133 °C. ^1^H NMR (600 MHz, CDCl_3_) δ 4.19 (s_br_, 2H), 5.38 (s_br_, 1H), 7.46–7.48 (m, 2H), 7.51–7.53 (m, 2H). ^13^C NMR (151 MHz, CDCl_3_) δ 43.8, 118.3 (q, ^1^*J*_C-F_ = 275.1 Hz, CF_3_), 120.6, 125.9, 131.9, 136.9 (q, ^2^*J*_C-F_ = 37.5 Hz, C(3)), 139.0, 157.8 (C=O). ^19^F NMR (565 MHz, CDCl_3_) δ −70.63 (s, CF_3_). IR (neat) *ν* 3295, 1692 and 1678 (C=O), 1487, 1349, 1316, 1193, 1137, 1059, 1010 cm^−1^. (−)-ESI-MS (*m*/*z*): 321.9 (88, [M{^81^Br}−H]^−^), 319.9 (100, [M{^79^Br}−H]^−^). Anal. Calcd (%) for C_10_H_7_BrF_3_N_3_O (322.1): C 37.29, H 2.19, N 13.05; found: C 37.28, H 2.10, N 13.13.

(*S*)-5-Methyl-1-(*p*-tolyl)-3-(trifluoromethyl)-4,5-dihydro-1,2,4-triazin-6(1*H*)-one (**11d**): CC (SiO_2_, DCM gradient DCM/EtOAc 6:1); colorless solid, 396 mg (73%), mp = 160–162 °C; [α]_D_^20^ = −14.9 (*c* = 0.0024, CHCl_3_). ^1^H NMR (600 MHz, CDCl_3_) δ 1.55 (d, *J* = 6.8 Hz, 3H), 2.35 (s, 3H), 4.29 (q_br_, *J* ≈ 6.8 Hz, 1H), 5.15 (s_br_, 1H), 7.19–7.21 (m, 2H), 7.38–7.40 (m, 2H). ^13^C NMR (151 MHz, CDCl_3_) δ 19.6, 21.2, 50.0, 118.4 (q, ^1^*J*_C-F_ = 274.9 Hz, CF_3_), 124.7, 129.4, 136.4 (q, ^2^*J*_C-F_ = 37.5 Hz, C(3)), 137.2, 137.9, 161.5 (C=O). ^19^F NMR (565 MHz, CDCl_3_) δ −70.76 (s, CF_3_). IR (neat) *ν* 3273, 1651 (C=O), 1513, 1361, 1286, 1193, 1137, 1096 cm^−1^. ESI-MS (*m*/*z*): 294.2 (19, [M+Na]^+^), 272.3 (100, [M+H]^+^). Anal. Calcd (%) for C_12_H_12_F_3_N_3_O (271.2): C 53.14, H 4.46, N 15.49; found: C 53.18, H 4.61, N 15.31.

5-Phenyl-1-(*p*-tolyl)-3-(trifluoromethyl)-4,5-dihydro-1,2,4-triazin-6(1*H*)-one (*rac*-**11e**): CC (SiO_2_, DCM); colorless solid, 530 mg (80%). ^1^H NMR (600 MHz, CD_3_OD) δ 2.36 (s, 3H), 5.25 (s_br_, 1H), 7.20–7.22 (m, 2H), 7.31–7.33 (m, 2H), 7.37–7.39 (m, 1H), 7.42–7.46 (m, 4H). NMR data are in accordance with the literature [35].

Methyl (*S*)-[6-oxo-1-(*p*-tolyl)-3-(trifluoromethyl)-4,5-dihydro-1,2,4-triazin-5(1*H*)-yl]acetate (**11f**): CC (SiO_2_, DCM gradient DCM/EtOAc 4:1); pale yellow solid, 434 mg (66%), mp = 120–122 °C; [α]_D_^20^ = −47.3 (*c* = 0.00075, CHCl_3_). ^1^H NMR (600 MHz, CDCl_3_) δ 2.35 (s, 3H), 2.85 (dd, *J* = 10.5, 17.6 Hz, 1H), 3.22 (dd, *J* = 2.7, 17.6 Hz, 1H), 3.78 (s, 3H), 4.61 (pseudo-dt, *J* ≈ 2.3, 10.5 Hz, 1H), 6.03 (s_br_, 1H), 7.19–7.21 (m, 2H), 7.38–7.40 (m, 2H). ^13^C NMR (151 MHz, CDCl_3_) δ 21.2, 37.2, 50.7, 52.6, 118.3 (q, ^1^*J*_C-F_ = 275.1 Hz, CF_3_), 124.6, 129.5, 136.4 (q, ^2^*J*_C-F_ = 37.5 Hz), 137.4, 137.7, 159.3 (C=O), 171.9 (C=O). ^19^F NMR (565 MHz, CDCl_3_) δ −70.78 (s, CF_3_). IR (neat) *ν* 3396, 1718 (C=O), 1685 (C=O), 1502, 1409, 1372, 1338, 1267, 1204, 1195, 1141, 1092 cm^−1^. ESI-MS (*m*/*z*): 352.2 (58, [M+Na]^+^), 330.3 (100, [M+H]^+^). Anal. Calcd (%) for C_14_H_14_F_3_N_3_O_3_ (329.3): C 51.07, H 4.29, N 12.76; found: C 51.01, H 4.29, N 12.61.

(*S*)-1-(4-Chlorophenyl)-5-methyl-3-(trifluoromethyl)-4,5-dihydro-1,2,4-triazin-6(1*H*)-one (**11g**): CC (SiO_2_, DCM gradient DCM/EtOAc 6:1); pale yellow solid, 449 mg (77%), mp = 163–164 °C; [α]_D_^20^ = −13.5 (*c* = 0.026, CHCl_3_). ^1^H NMR (600 MHz, CDCl_3_) δ 1.54 (d, *J* = 6.8 Hz, 3H), 4.28 (q_br_, *J* ≈ 6.8 Hz, 1H), 5.34 (s_br_, 1H), 7.35–7.37 (m, 2H), 7.50–7.53 (m, 2H). ^13^C NMR (151 MHz, CDCl_3_) δ 19.5, 50.0, 118.3 (q, ^1^*J*_C-F_ = 275.1 Hz, CF_3_), 125.7, 128.8, 132.6, 136.9 (q, ^2^*J*_C-F_ = 37.6 Hz), 138.8, 161.5 (C=O). ^19^F NMR (565 MHz, CDCl_3_) δ −70.80 (s, CF_3_). IR (neat) *ν* 3247, 1681–1664 (C=O), 1491, 1346, 1223, 1189, 1133, 1081 cm^−1^. ESI-MS (*m*/*z*): 292.2 (100, [M+H]^+^). Anal. Calcd (%) for C_11_H_9_ClF_3_N_3_O (291.7): C 45.30, H 3.11, N 14.41; found: C 45.36, H 3.00, N 14.25.

1-(4-Chlorophenyl)-5-phenyl-3-(trifluoromethyl)-4,5-dihydro-1,2,4-triazin-6(1*H*)-one (**11h**): CC (SiO_2_, DCM gradient DCM/EtOAc 4:1); pale yellow solid, 544 mg (77%), mp = 148–149 °C. ^1^H NMR (600 MHz, CDCl_3_) δ 5.09 (s, 1H), 5.95 (s_br_, 1H), 7.30–7.48 (m, 9H). ^13^C NMR (151 MHz, CDCl_3_) δ 57.9, 118.4 (q, ^1^*J*_C-F_ = 275.2 Hz, CF_3_), 125.7, 126.7, 128.8, 129.4, 129.5, 132.7, 136.4 (q, ^2^*J*_C-F_ = 37.4 Hz), 137.9, 138.7, 159.3 (C=O). ^19^F NMR (565 MHz, CDCl_3_) δ −70.43 (s, CF_3_). IR (neat) *ν* 3258, 1684–1651 (C=O), 1491, 1342, 1286, 1193, 1152, 1081 cm^−1^. ESI-MS (*m*/*z*): 354.1 (100, [M+H]^+^). Anal. Calcd (%) for C_16_H_11_ClF_3_N_3_O (353.7): C 54.33, H 3.13, N 11.88; found: C 54.23, H 3.30, N 11.60.

### 4.2. Biological Analyses—General Methods

#### 4.2.1. Antimicrobial Activity

The antifungal and antibacterial activities of the investigated compounds **7a**–**7h** were evaluated by determining their minimum inhibitory concentrations (MICs) using a 96-well microdilution assay against four fungal species (*Candida albicans*, *Candida glabrata*, *Trichophyton rubrum*, and *Epidermophyton floccosum*) and five bacterial species (*Staphylococcus aureus*, *Staphylococcus epidermidis*, *Proteus vulgaris*, *Klebsiella pneumoniae*, and *Escherichia coli*). The experiments were performed in accordance with the guidelines of the European Committee on Antimicrobial Susceptibility Testing (EUCAST) for MIC_50_ determination in yeasts, dermatophytes, and bacteria (EUCAST, 08.09.2020; EUCAST, 01.01.2022; EUCAST, 13.10.2023).

Preparation of Yeast Cultures: *Candida albicans* was streaked onto Sabouraud dextrose agar (SDA) plates and incubated at 35 °C for 24 h. A single colony was then transferred into 5 mL of liquid Sabouraud medium and incubated for 2 h at 28 °C with shaking at 120 rpm. The inoculum density was adjusted to 0.5 McFarland units using a densitometer. According to the EUCAST recommendations, the suspension was diluted tenfold in RPMI 1640 medium to obtain a final inoculum density of 2–5 × 10^5^ CFU/mL. The same procedure was followed for *Candida glabrata*.

Preparation of Dermatophyte Cultures: Dermatophytes were cultured on Sabouraud agar slants for 21 days at 28 °C. To separate conidia from the mycelium, cultures were washed with 5 mL of liquid Sabouraud medium, gently scraped with a sterile inoculating loop, and filtered through a 40 μm cell strainer (Falcon^®^, 40 μm Cell Strainer). After centrifugation (5 min, 4 °C, 4000 rpm), the supernatant was discarded, and the pellet was inoculated into 20 mL of YG medium. Following incubation (2 h, 37 °C, 140 rpm), the cultures were centrifuged again (5 min, 4 °C, 4000 rpm) to obtain a pellet containing germinating conidia, which were subsequently resuspended in 2 mL of sterile distilled water. The inoculum was then diluted to a final concentration of 2–5 × 10^5^ CFU/mL. This procedure was used to prepare inocula of *Trichophyton rubrum* and *Epidermophyton floccosum*.

Preparation of Bacterial Cultures: Bacterial strains were streaked onto appropriate agar media and incubated at 37 °C for 24 h to obtain isolated colonies. A single colony was subsequently transferred into 30 mL of Mueller–Hinton (MH) broth and incubated overnight at 37 °C with shaking at 180 rpm under aerobic conditions. Bacterial cultures were diluted with fresh MH broth to obtain a final inoculum density of 2–5 × 10^5^ CFU/mL. This procedure was applied to prepare cultures of *Staphylococcus aureus*, *Staphylococcus epidermidis*, *Proteus vulgaris*, *Klebsiella pneumoniae*, and *Escherichia coli*.

Determination of Minimum Inhibitory Concentrations (MICs): The investigated [1,2,4]-triazine derivatives **7a**–**7h** were dissolved in dimethyl sulfoxide (DMSO) to obtain a stock concentration of 12.8 mg/mL. The final DMSO concentration did not exceed 1% and had no significant effect on microbial growth. Initially, two-fold serial dilutions of the tested compounds were prepared in 96-well microplates to obtain final concentrations ranging from 0.0625 to 128 mg/L. Row A served as the negative control, containing only culture medium without microorganisms. Row B served as the positive control, containing culture medium supplemented with the microbial inoculum. The prepared microbial suspensions (2–5 × 10^5^ CFU/mL) were added to each well except those in row A. The plates were incubated at 37 °C, with incubation times depending on the microorganism tested: 2 days for *Candida* species, 3 days for *Trichophyton rubrum*, 5 days for *Epidermophyton floccosum*, and 24 h for all bacterial species. Following incubation, the growth of *Candida albicans*, *Candida glabrata*, and bacterial cultures was assessed spectrophotometrically by measuring the optical density at 595 nm (OD_595_). In the case of dermatophytes, the antifungal activity of the tested compounds was evaluated microscopically for each well. The micrographs presented in the main text and the ESI are intended to illustrate morphological changes rather than to provide a quantitative assessment of fungal growth. They represent typical fields of view from independent biological replicates. Growth inhibition was assessed by microscopic evaluation of the entire culture well rather than from individual microscopic images. Slight differences in mycelial density between images are expected because of the inherent variability in spore germination, particularly in *E. floccosum*. The obtained MIC_50_ values were compared with those of the reference antimicrobial agents, including the antifungal drugs clotrimazole and itraconazole (Merck) and the antibacterial agents vancomycin and ciprofloxacin (Merck). Each compound was evaluated in two independent experiments.

#### 4.2.2. Cytotoxic Activity

Cell lines and cell culture: The promyelocytic leukemia HL-60, breast cancer adenocarcinoma MCF-7, and lung cancer A549 cell lines were purchased from the European Collection of Cell Cultures (ECACC). The prostate carcinoma DU-145 cell line was a gift from dr Kamila Domińska (Department of Comparative Endocrinology, Medical University of Lodz) and was obtained from the American Type Culture collection (ATCC). Leukemia and DU-145 cells were cultured in Roswell Park Memorial Institute (RPMI) 1640 plus GlutaMax I medium (Gibco/Life Technologies, Carlsbad, CA, USA). For DU-145 cells medium was additionally supplemented with 1 mM sodium pyruvate and 10 mM HEPES Buffer (both from Gibco/Life Technologies, Carlsbad, CA, USA). MCF-7 cells were maintained in Minimum Essential Medium Eagle (Sigma Aldrich, St. Louis, MO, USA). supplemented with 2 mM glutamine and MEM nonessential amino-acid solution (both from Sigma Aldrich, St. Louis, MO, USA). A549 cells were grown in Kaighn’s Modification of Ham’s F-12 Medium (F-12K Medium) from ATCC. All culture media mentioned above were supplemented with 10% heat-inactivated fetal bovine serum (Biological Industries, Beit-Haemek, Israel) and antibiotics (100 U/mL penicillin and 100 μg/mL streptomycin) (Sigma-Aldrich, St. Louis, MO, USA). Human umbilical vein endothelial cells (HUVECs) were purchased from ATCC and cultured using the EGM-2 Endothelial Medium BulletKit (Lonza, Walkersville, MD, USA). All cells were maintained at 37 °C in 5% CO_2_ atmosphere and grown until 80% confluent.

Tested compounds were dissolved in sterile DMSO and then diluted in culture medium. Final DMSO concentration in culture was below 0.1% *v*/*v*. At the used concentration, DMSO had no influence on the evaluated parameters. Carboplatin used as a reference compound was obtained form (Sigma-Aldrich, St. Louis, MO, USA).

In vitro cytotoxicity assay: The MTT (3-(4, 5-dimethylthiazol-2-yl)-2,5-diphenyltetrazolium bromide) assay was performed according to a previously described procedure [45]. Cells were seeded into 24-well plates at a density of 8 × 10^4^ cells/mL (HL-60, MCF-7, DU-145, HUVECs) or 5 × 10^4^ cells/mL for A549 cells and allowed to adhere for 24 h. The cells were then cultured for 48 h in the presence of various concentrations of the tested compounds before incubation with MTT solution (5 mg/mL in phosphate-buffered saline) for 2 h. Then, the plates were centrifuged and the supernatant was discarded. Dimethyl sulfoxide (DMSO; 1 mL) was added to each well to dissolve the blue formazan product, whose absorbance was measured at 560 nm using a FlexStation 3 Multi-Mode Microplate Reader (Molecular Devices, LLC, CA, USA). The untreated cells were used as control. The concentration inhibiting cell viability by 50% (IC_50_) was calculated from concentration–response curves. Data are presented as mean ± SEM of three independent experiments.

## 5. Conclusions

A series of 3-trifluoromethyl-1,2,4-triazin-6(1*H*)-ones was designed and synthesized through a concise two-step strategy involving (3+3)-cycloaddition of in situ-generated CF_3_-nitrile imines with amino acid esters, followed by DDQ-mediated chemoselective oxidation. The devised methodology provides efficient access to structurally diverse fluorinated 1,2,4-triazines starting from inexpensive and readily available starting materials. Biological evaluation against representative fungal and bacterial pathogens, together with cytotoxicity studies toward four human cancer cell lines and normal HUVEC cells, revealed a distinct selectivity profile. The synthesized compounds displayed promising antifungal activity, whereas their antibacterial and antiproliferative effects were generally weak. The highest antifungal potency was observed for C(5)-unsubstituted 1,2,4-triazines, particularly the *para*-bromophenyl analogue, which exhibited the broadest antimicrobial spectrum and inhibited the growth of *Candida albicans*, *Candida glabrata*, dermatophytes, and several bacterial strains. In addition, an unusual biphasic concentration-dependent response, manifested by growth stimulation at subinhibitory concentrations and growth inhibition at higher concentrations, was observed for dermatophytes, representing a hormesis effect rarely reported for fungi. Structure–activity relationship analysis indicates that the unsubstituted C(5) position and the corresponding imine functionality play a crucial role in determining biological activity. Although the most active derivatives also exhibited measurable cytotoxicity toward normal cells, the results identify trifluoromethylated 1,2,4-triazin-6(1*H*)-ones as promising lead structures for the development of new antifungal agents and provide valuable guidance for further structural optimization.

## Figures and Tables

**Table 1:** Oxidation of the N(4)–C(5) bond in **11a**: reaction conditions.

Entry	Oxidant	Solvent	Temperature	Time	Conversion [Yield]
1	MnO_2_ (20 equiv.)	DCM	rt	24 h	-
2	MnO_2_ (20 equiv.)	THF	rt	24 h	-
3	MnO_2_ (20 equiv.)	THF	60 °C	24 h	-
4	DDQ (1.25 equiv.)	EtOAc	77 °C	1 h	~40%
5	DDQ (1.50 equiv.)	EtOAc	77 °C	24 h	100% [79%] ^1^
6	K_3_[Fe(CN)_6_] (6.0 equiv.)	DCM ^2^	rt	24 h	100% [69%] ^1^

^1^ Isolated yield; ^2^ Reaction carried out in the presence of K_2_CO_3_ (0.5M in H_2_O) and [Et_4_N]Br (10 mol%).

**Table 2:** In vitro cytotoxic activity of 1,2,4-triazin-6-ones **7a**–**7h** in µM (in mg/L in brackets).

Compound	IC_50_ [μM] ^1^				
	HL-60	MCF-7	A549	DU-145	HUVEC
**7a**	9.85 ± 0.11 (2.51)	45.75 ± 7.24 (11.68)	39.68 ± 0.85 (10.13)	78.36 ± 3.28 (20.00)	23.04 ± 1.72 (5.88)
**7b**	3.14 ± 0.02 (1.09)	8.05 ± 0.04 (2.80)	16.20 ± 0.90 (5.63)	37.61 ± 0.04 (13.06)	3.99 ± 0.15 (1.39)
**7c**	3.54 ± 0.03 (1.13)	16.25 ± 0.25 (5.20)	23.22 ± 0.73 (7.43)	62.89 ± 1.17 (20.13)	5.86 ± 0.14 (1.88)
**7d**	-	-	-	-	-
**7e**	-	-	-	-	-
**7f**	-	-	-	-	-
**7g**	-	-	-	-	-
**7h**	-	-	-	-	-
Carboplatin	3.80 ± 0.04 (1.41)	2.90 ± 0.10 (1.08)	-	105 ± 0.13 (37.12)	5.35 ± 0.05 (1.99)

^1^ Compound concentration required to inhibit metabolic activity by 50%. Values are expressed as mean ± SEM from concentration-response curves of at least three experiments.

## Data Availability

The original contributions presented in this study are included in the article/Supplementary Materials. Further inquiries can be directed to the corresponding author.

## Supplementary Materials

The following supporting information can be downloaded at: https://www.mdpi.com/article/10.3390/ijms27156796/s1.
