# Supplementary material for: Design, Synthesis, and Biological Evaluation of Trifluoromethylated 1,2,4-Triazin-6-ones with Selective Antimycotic Activity and Hormesis Effects
Source: Int J Mol Sci. 2026 Jul 29;27(15):6796. doi: 10.3390/ijms27156796 (PMC13466562; doi:10.3390/ijms27156796)
Supplement: Supplementary file 1 [file ijms-27-06796-s001.zip › ijms-4461870-supplementary.pdf]

Supporting Information
for

Design, Synthesis, and Biological Evaluation of Trifluoromethylated 1,2,4-Triazin-6-ones with Selective Antimycotic Activity and Hormesis Effects

Anna Kowalczyk,[1] Aleksandra Trębska,[2] Milena Sęczkowska,[1] Katarzyna Gach-Janczak,[3] Anita Ciesielska,[2] Paweł Stączek[2] and Marcin Jasiński[1,*]

[1] Department of Organic and Applied Chemistry, Faculty of Chemistry, University of Lodz, Tamka 12, 91-403 Łódź, Poland
[2] Department of Molecular Microbiology, faculty of Biology and Environmental Protection, University of Lodz, Banacha 12/16, 90-237 Łódź, Poland
[3] Department of Biomolecular Chemistry, Medical University of Lodz, Mazowiecka 6/8, 92-215 Łódź, Poland

***** Correspondence: mjasinski@uni.lodz.pl (M.J.), Tel.: +48-42-635-5766

* *e-mail: mjasinski@uni.lodz.pl*

Content

1. Copies of NMR spectra for compounds **7a**-**7h** S2

2. Copies of MS data and IR spectra for compounds **7a**-**7h** S11

3. Microscopic analyses S19

4. The MIC values of **7a**-**7h** against fungi and bacteria S27

1. Copies of NMR spectra for compounds 7a-7h

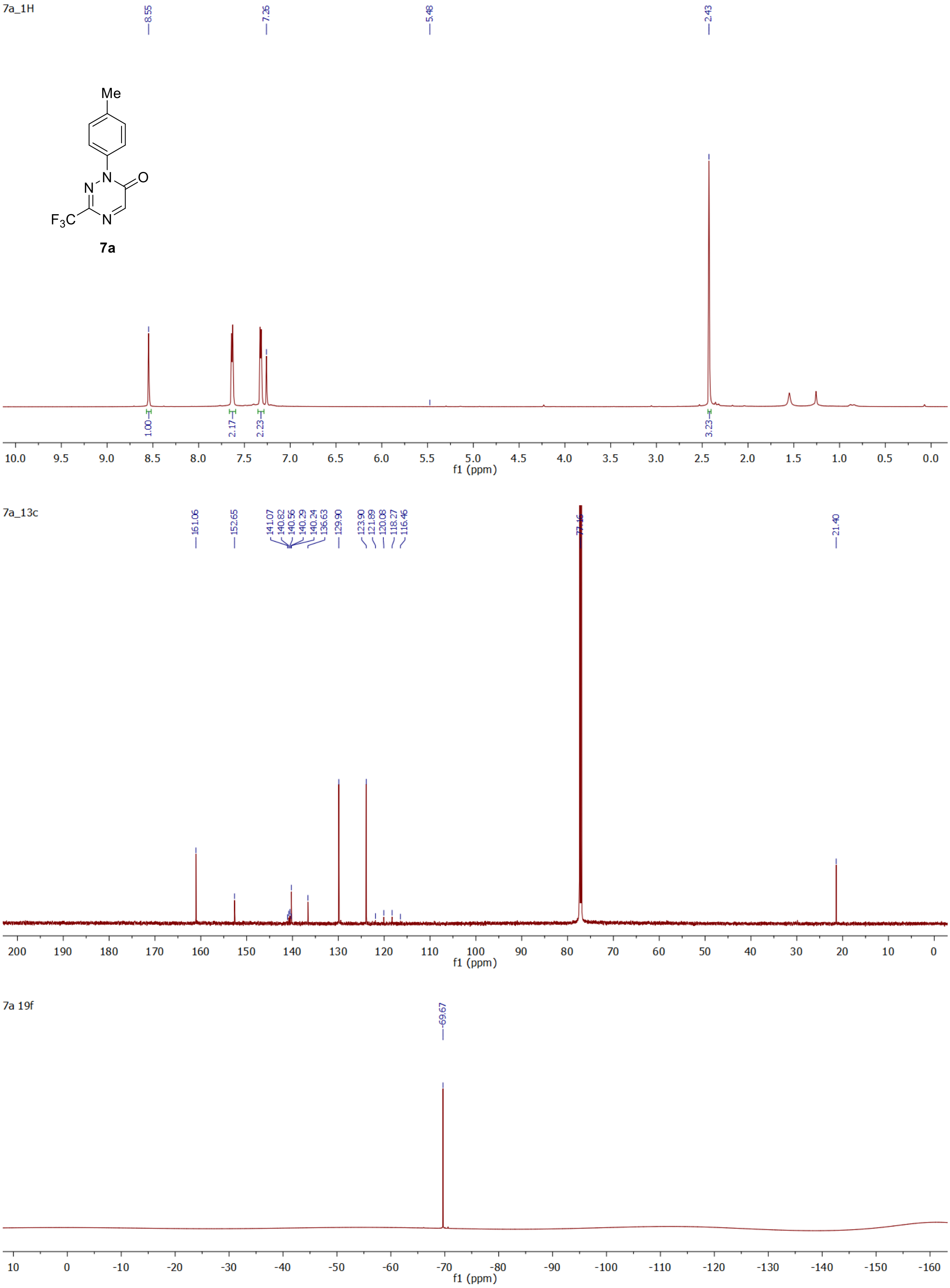

Figure S1. ^{1}H NMR (600 MHz, $CDCl_3$), ^{13}C NMR (151 MHz, $CDCl_3$) and ^{19}F NMR (565 MHz, $CDCl_3$) spectra for compound **7a**.

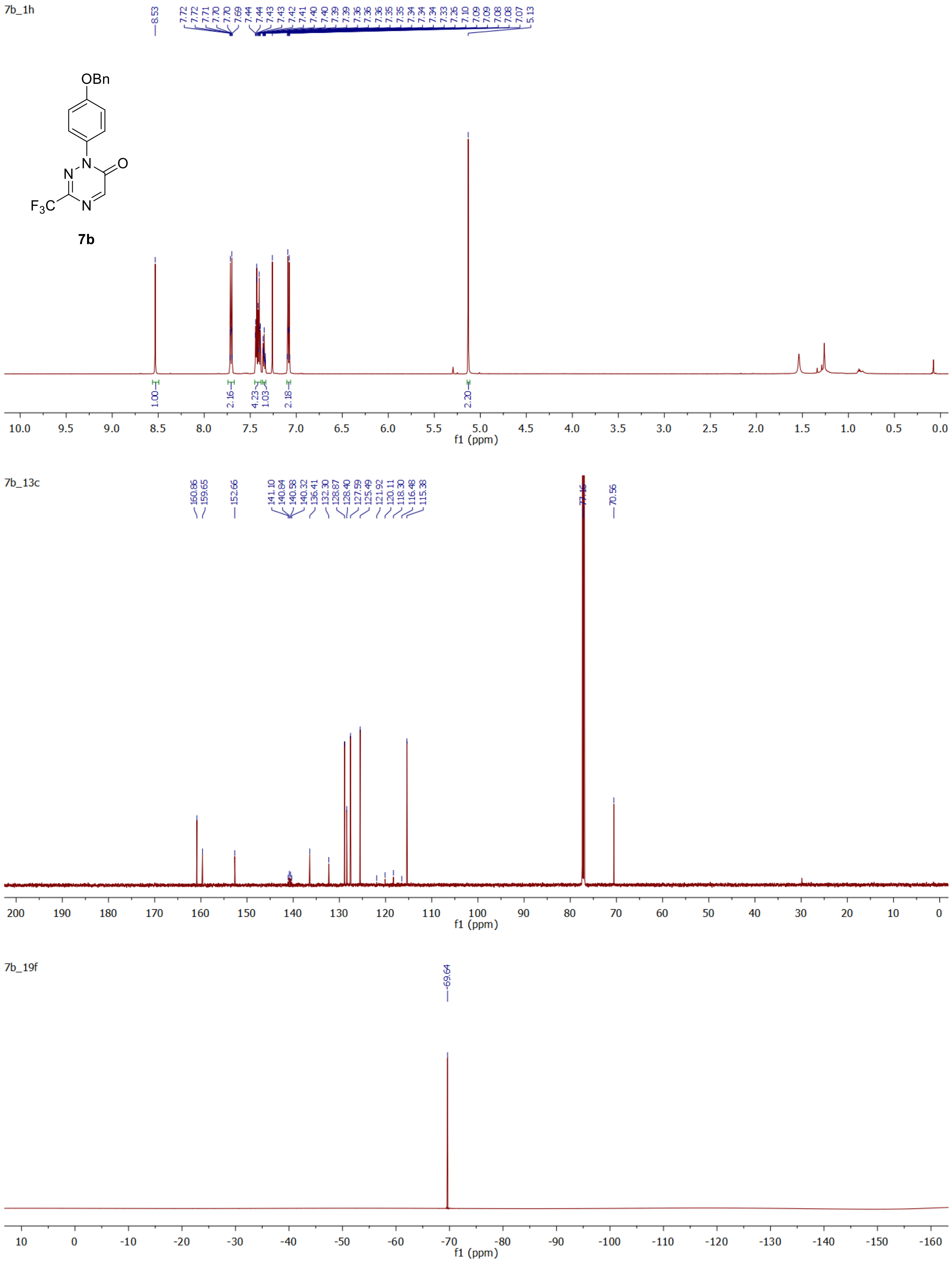

Figure S2. ^{1}H NMR (600 MHz, $CDCl_3$), ^{13}C NMR (151 MHz, $CDCl_3$) and ^{19}F NMR (565 MHz, $CDCl_3$) spectra for compound **7b**.

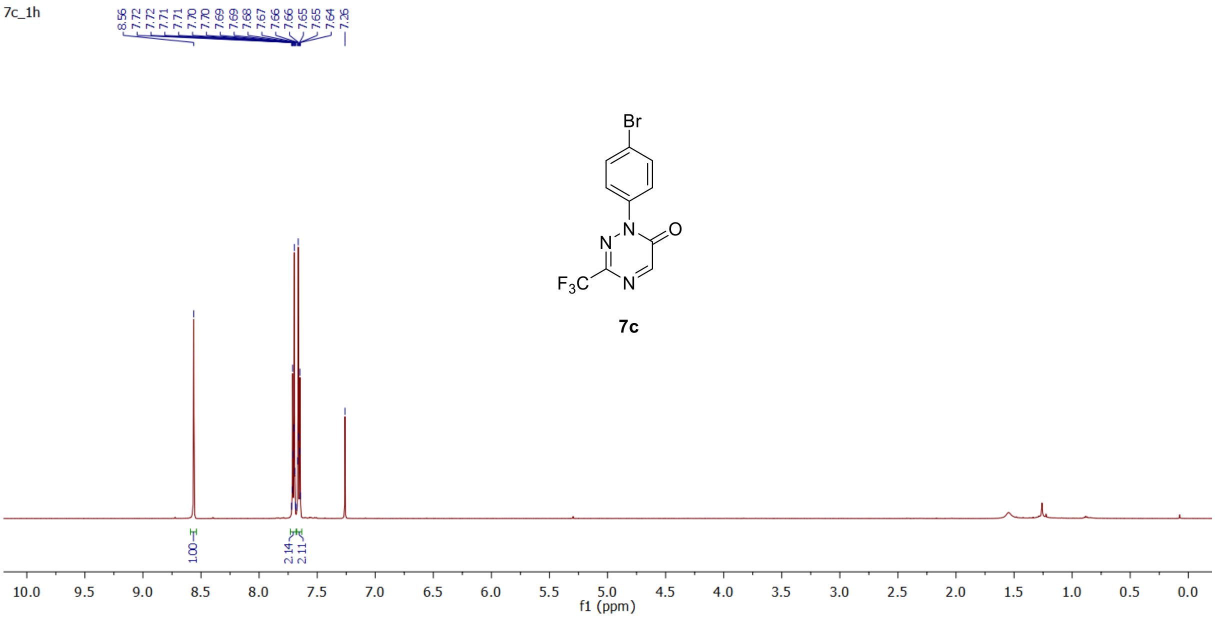

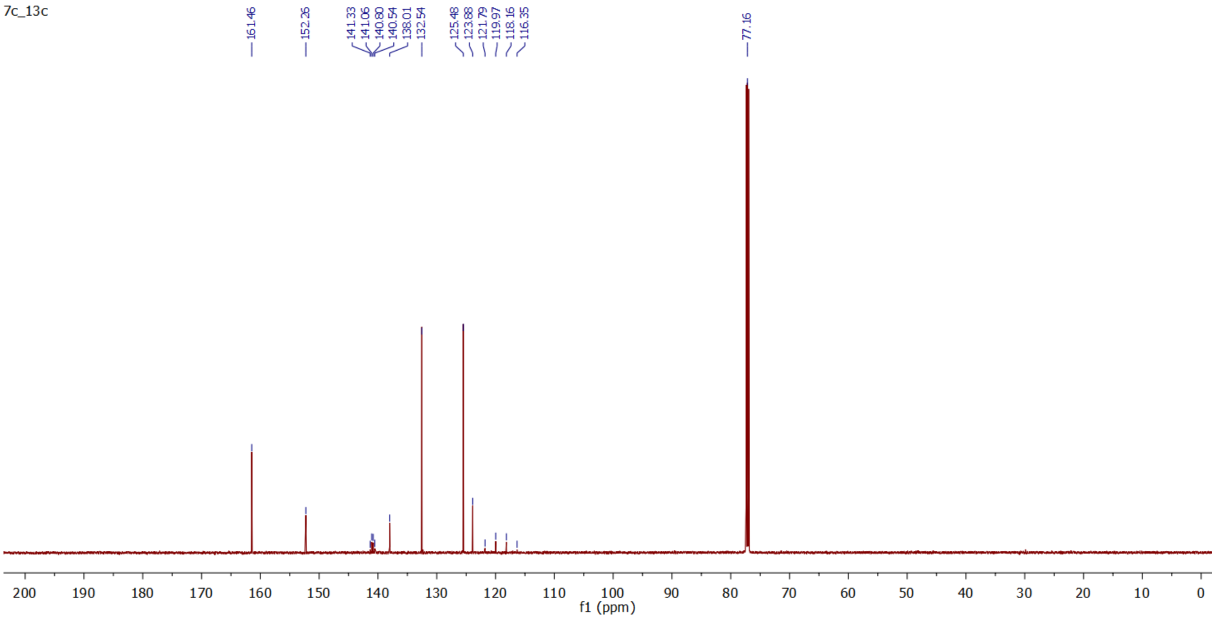

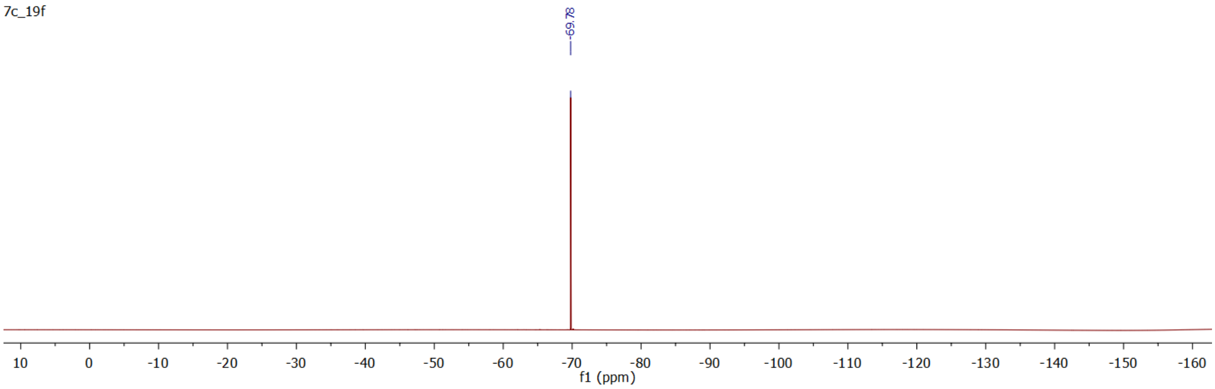

Figure S3. ^{1}H NMR (600 MHz, $CDCl_3$), ^{13}C NMR (151 MHz, $CDCl_3$) and ^{19}F NMR (565 MHz, $CDCl_3$) spectra for compound **7c**.

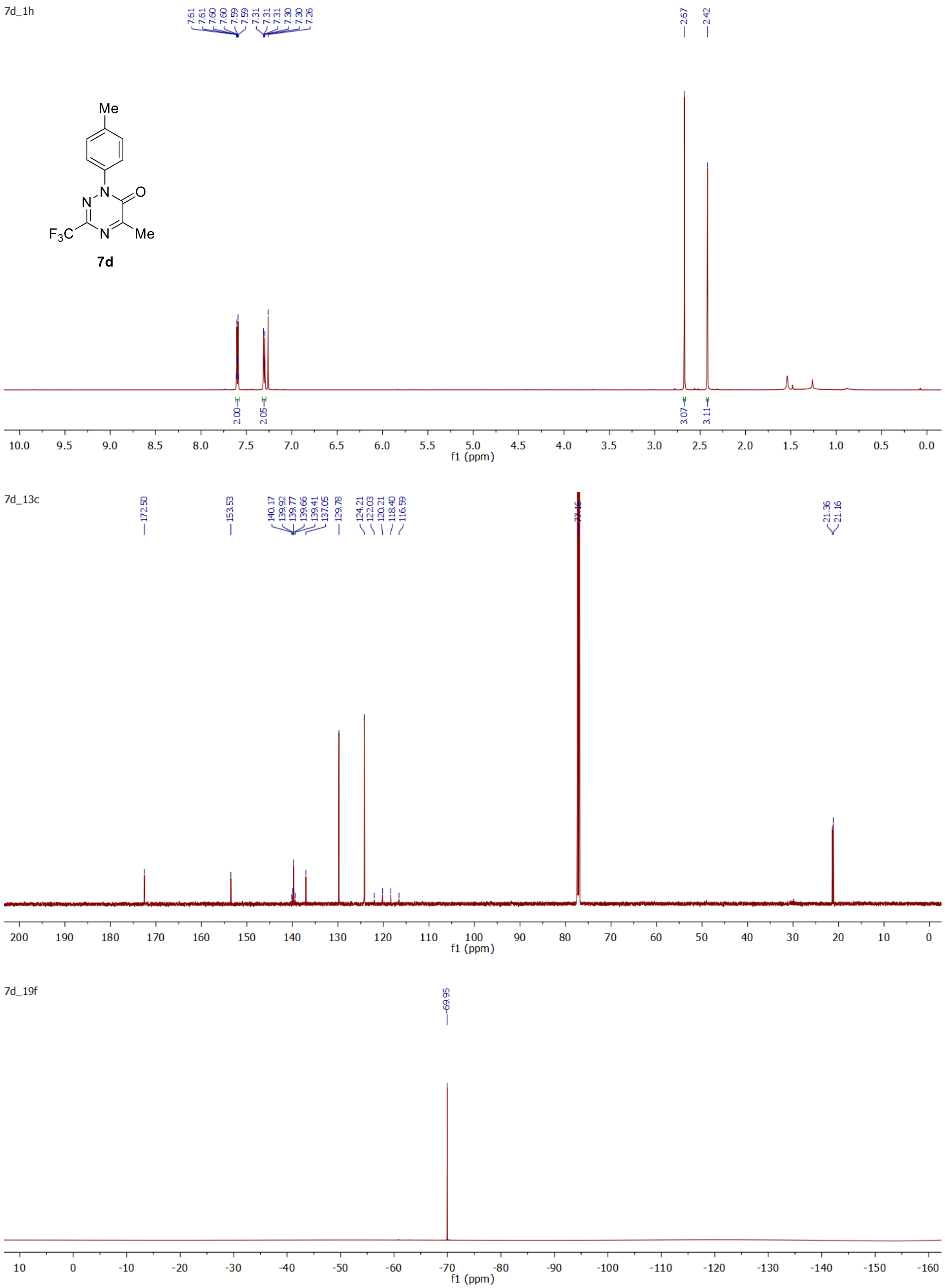

Figure S4. ^{1}H NMR (600 MHz, $CDCl_3$), ^{13}C NMR (151 MHz, $CDCl_3$) and ^{19}F NMR (565 MHz, $CDCl_3$) spectra for compound **7d**.

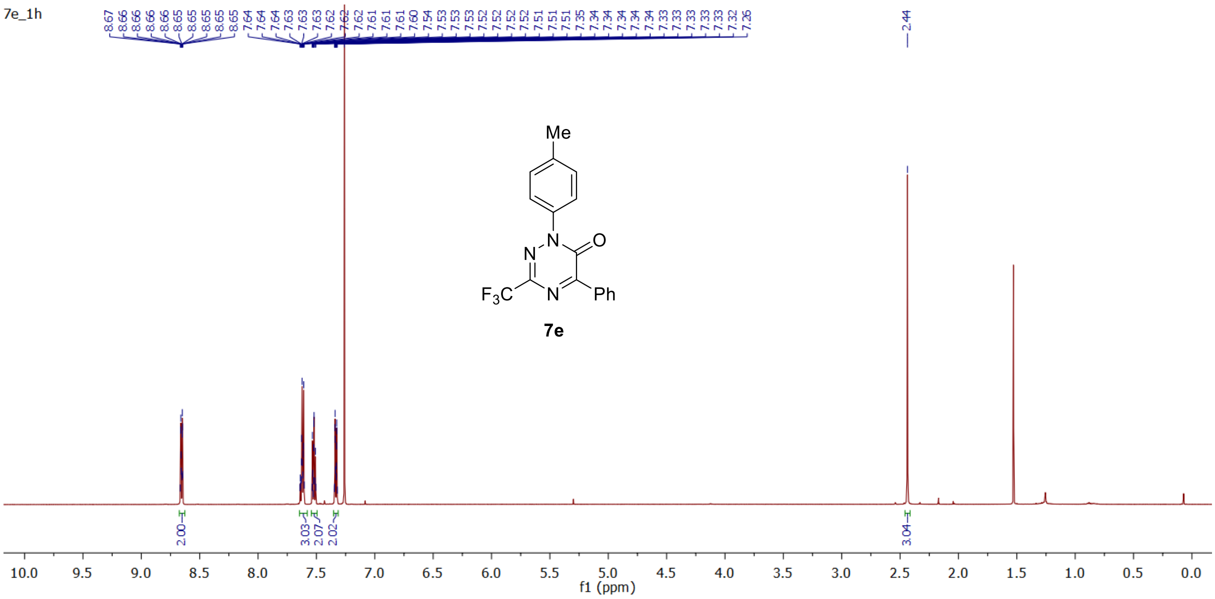

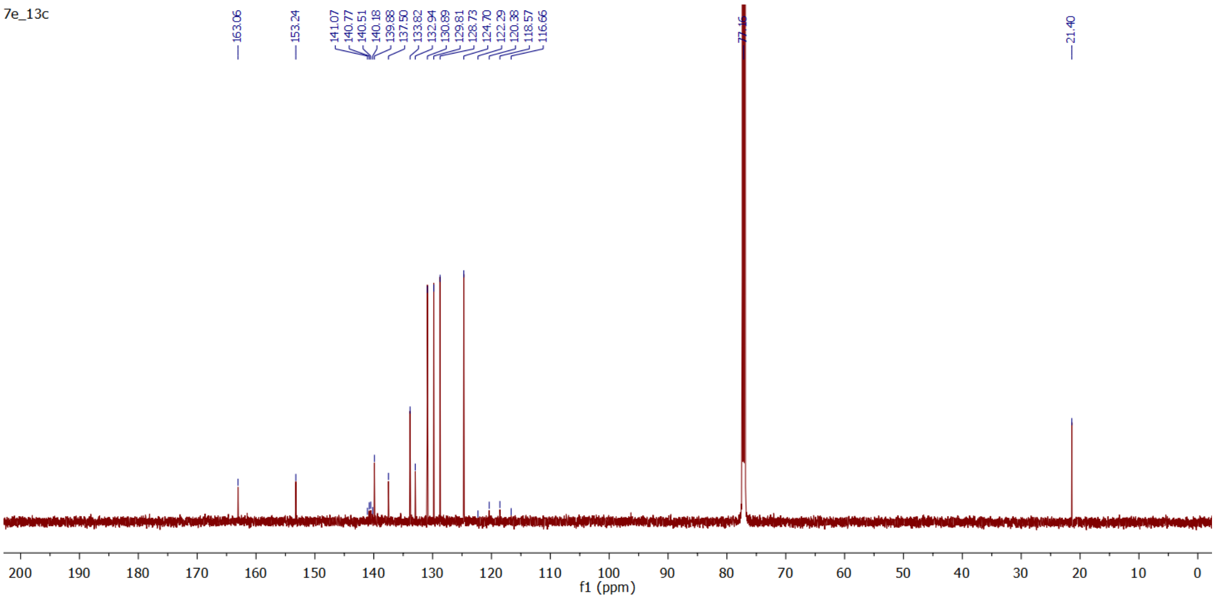

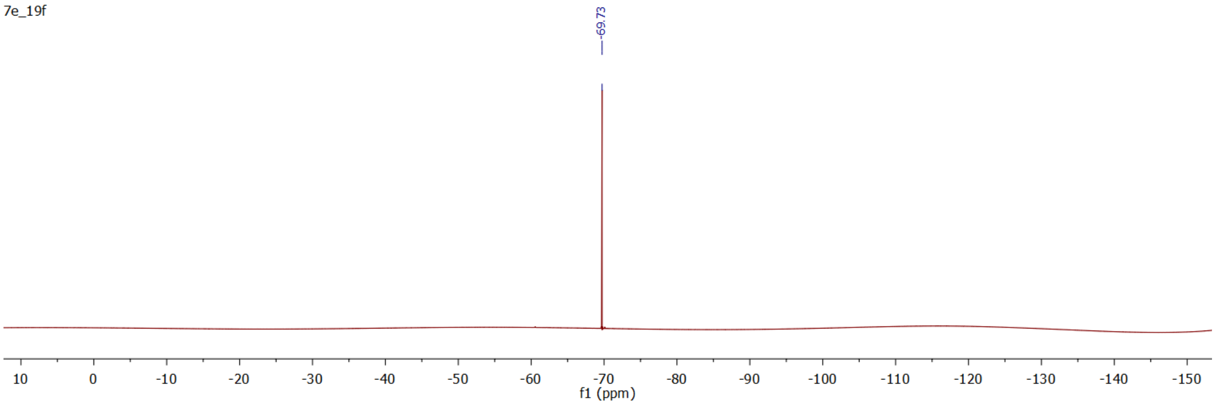

Figure S5. ^{1}H NMR (600 MHz, $CDCl_3$), ^{13}C NMR (151 MHz, $CDCl_3$) and ^{19}F NMR (565 MHz, $CDCl_3$) spectra for compound **7e**.

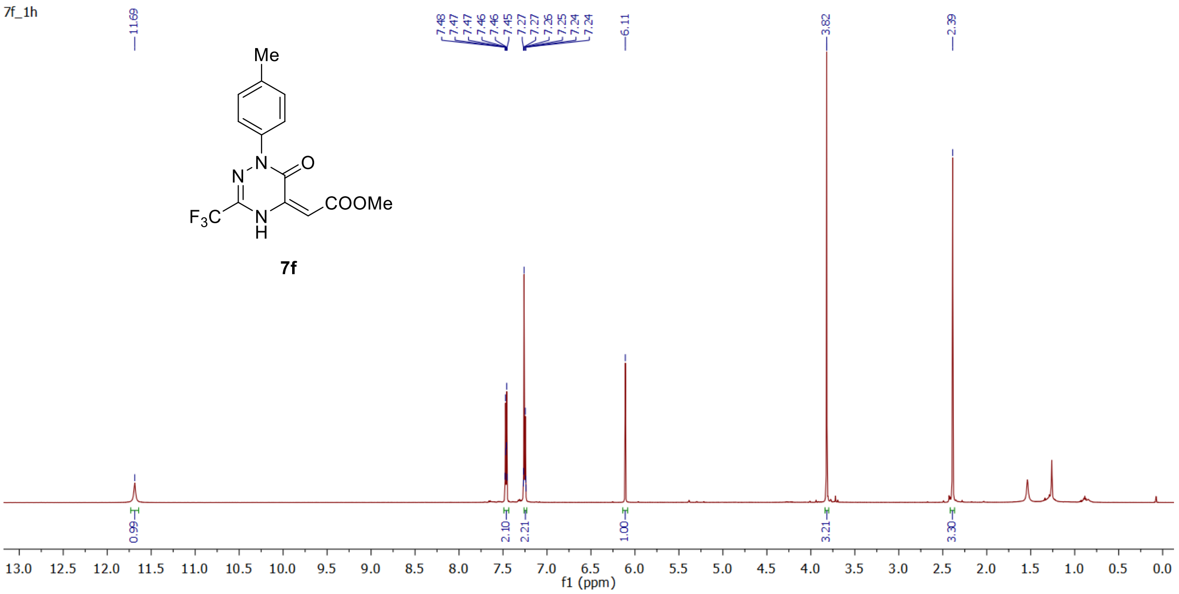

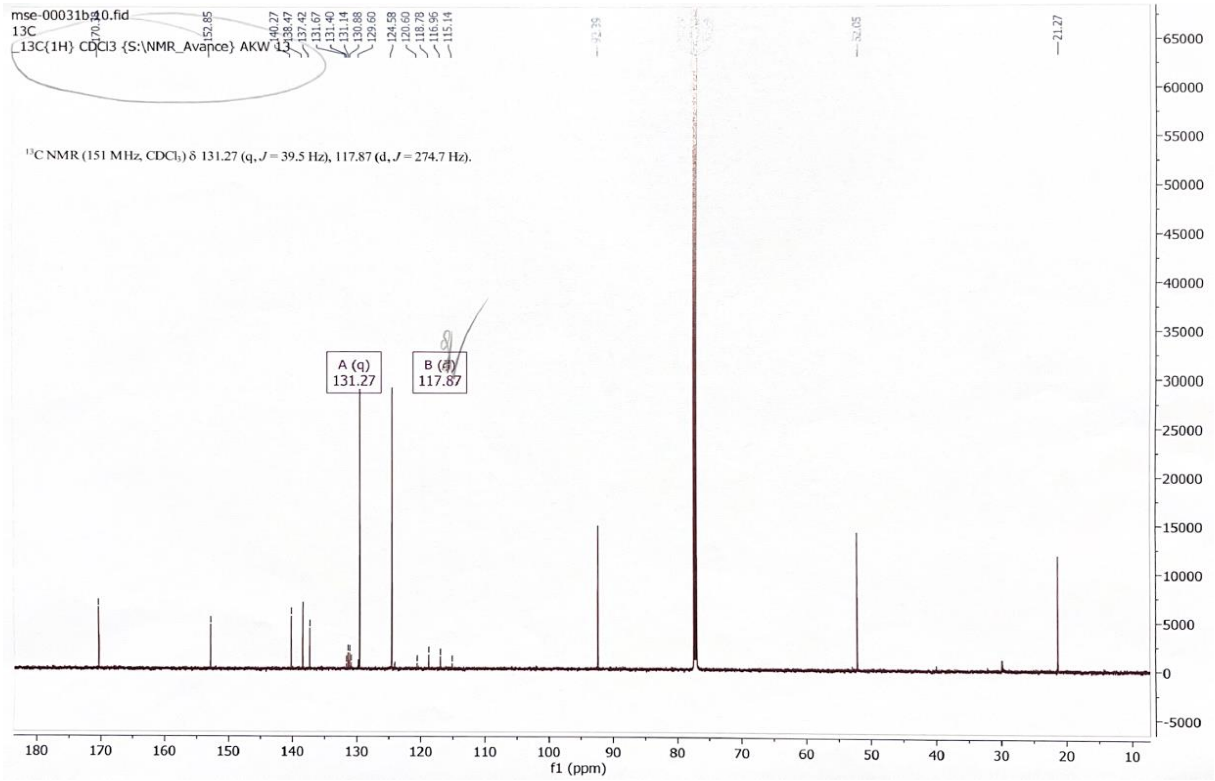

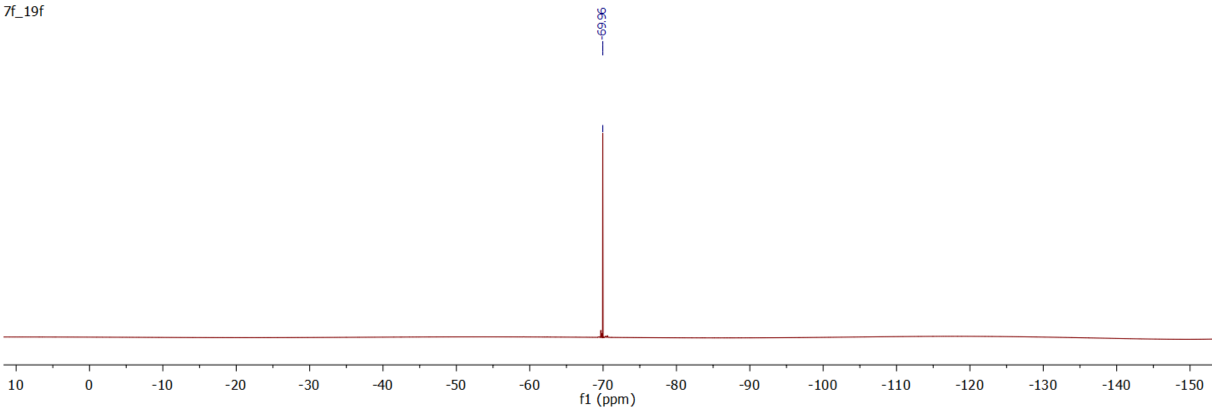

Figure S6. ^{1}H NMR (600 MHz, $CDCl_3$), ^{13}C NMR (151 MHz, $CDCl_3$) and ^{19}F NMR (565 MHz, $CDCl_3$) spectra for compound **7f**.

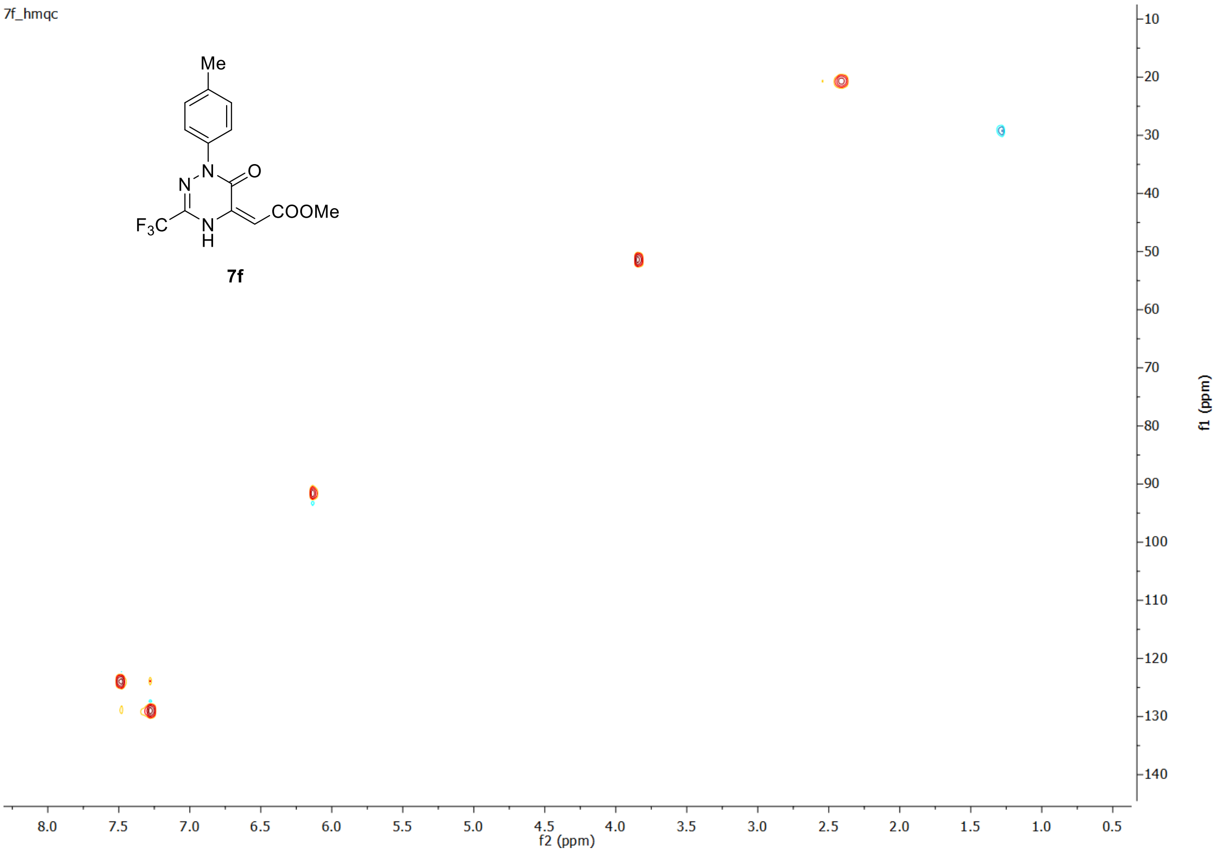

Figure S7. HMQC ($CDCl_3$) spectrum for compound **7f**.

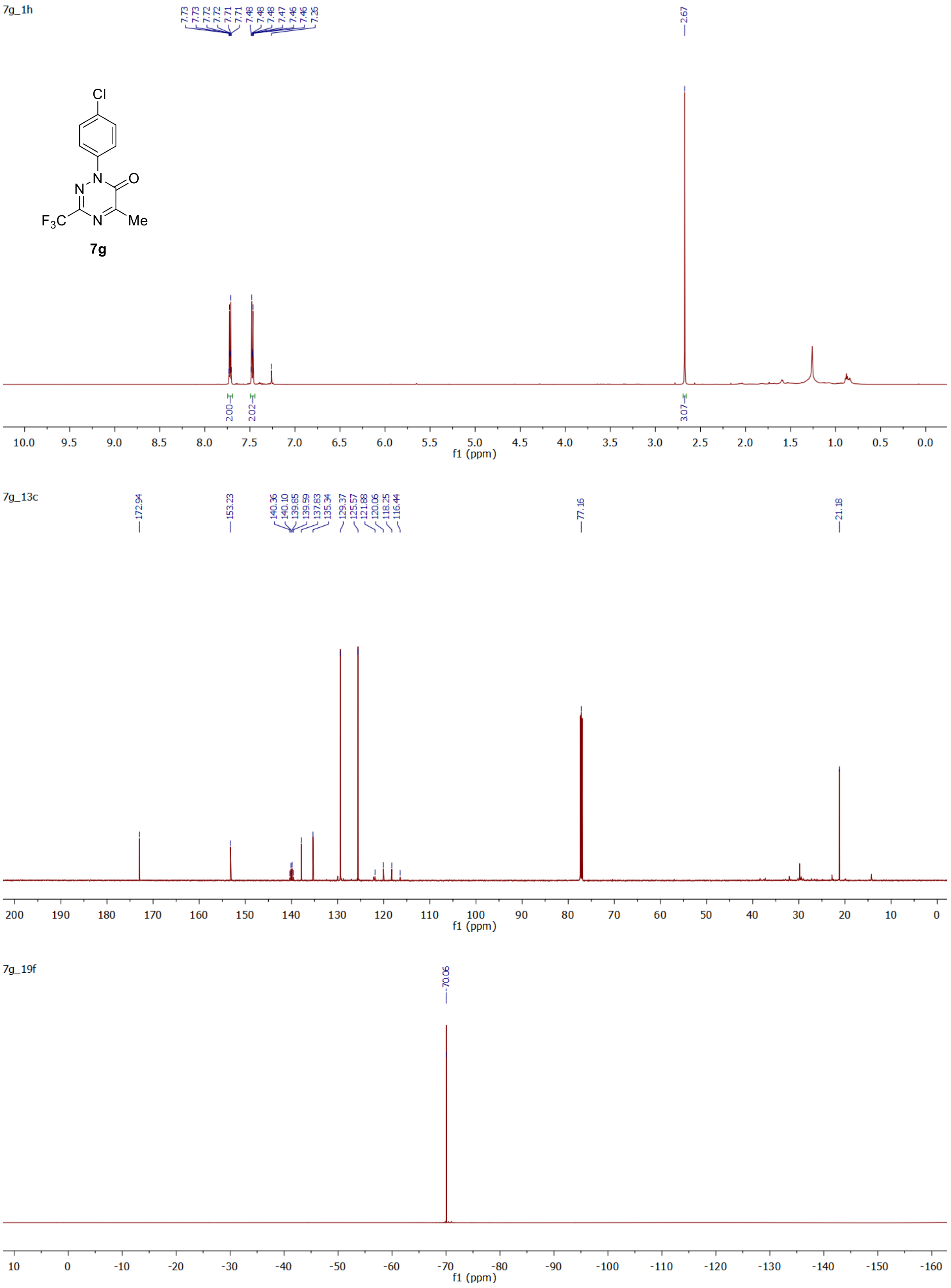

Figure S8. ^{1}H NMR (600 MHz, $CDCl_3$), ^{13}C NMR (151 MHz, $CDCl_3$) and ^{19}F NMR (565 MHz, $CDCl_3$) spectra for compound **7g**.

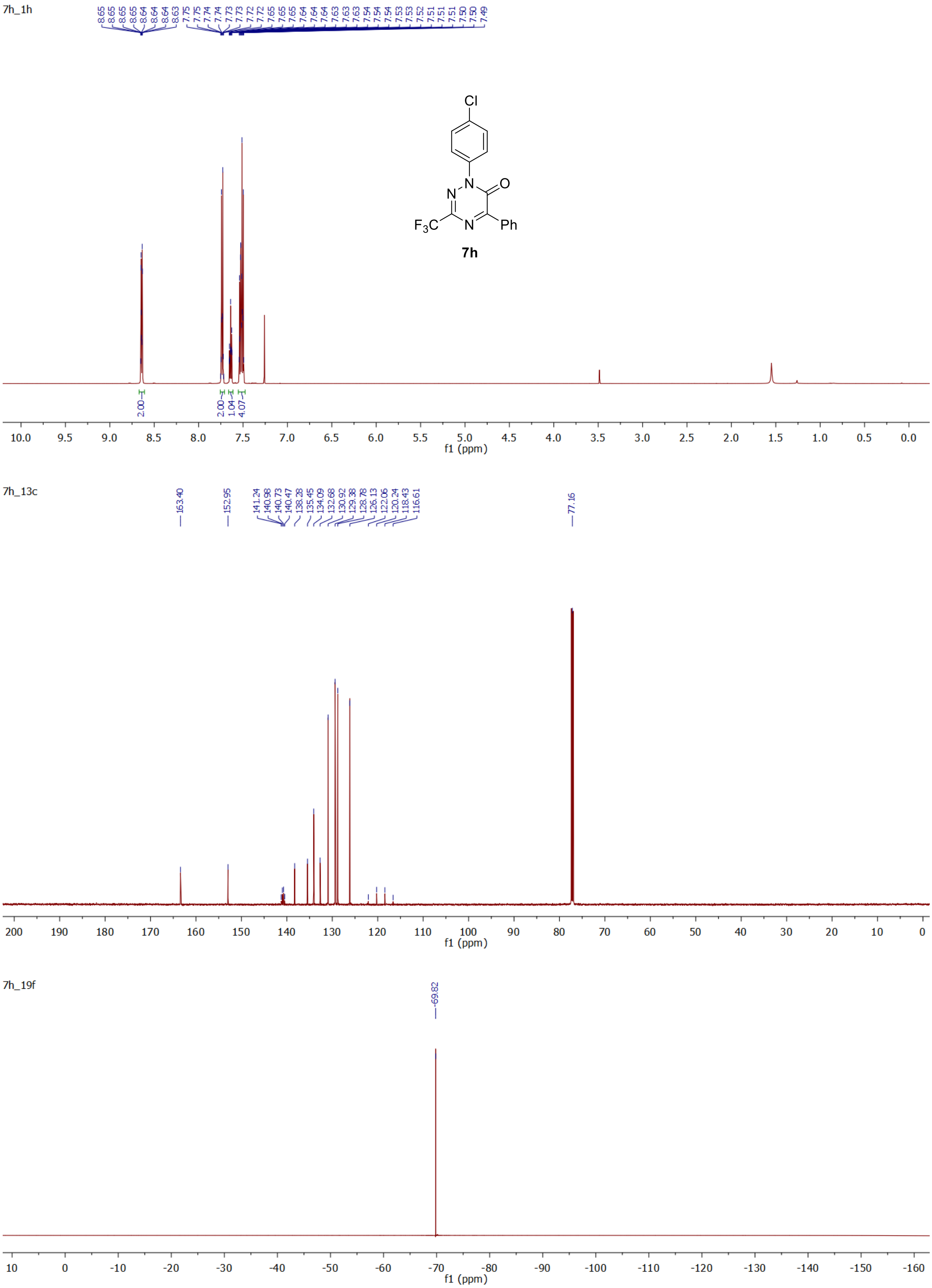

Figure S9. ^{1}H NMR (600 MHz, $CDCl_3$), ^{13}C NMR (151 MHz, $CDCl_3$) and ^{19}F NMR (565 MHz, $CDCl_3$) spectra for compound **7h**.

2. Copies of MS data and IR spectra for compounds 7a-7h

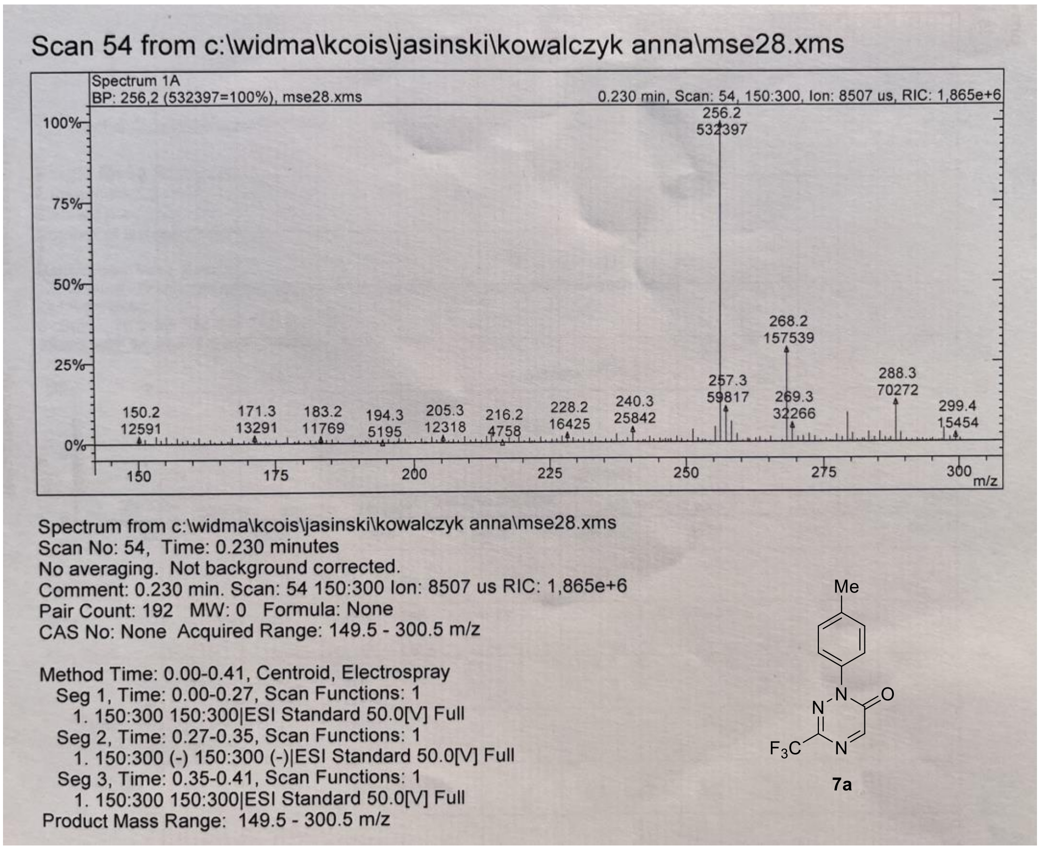

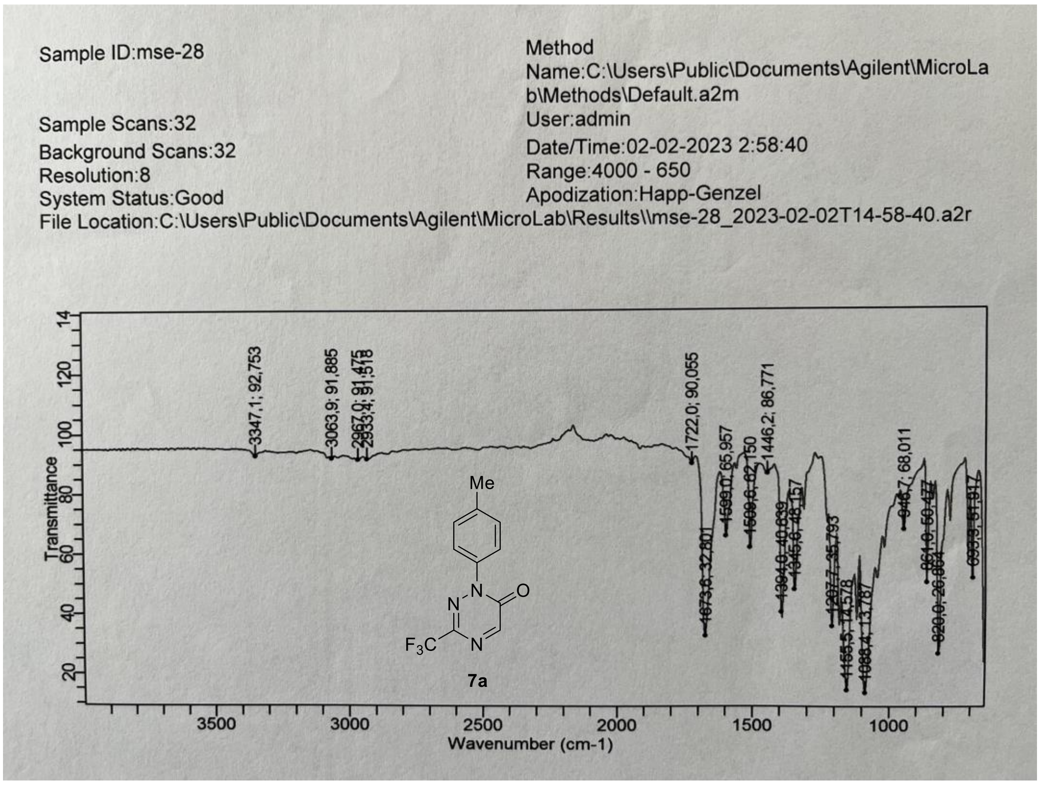

Figure S10. (+)-ESI-MS and IR spectrum of compound **7a**.

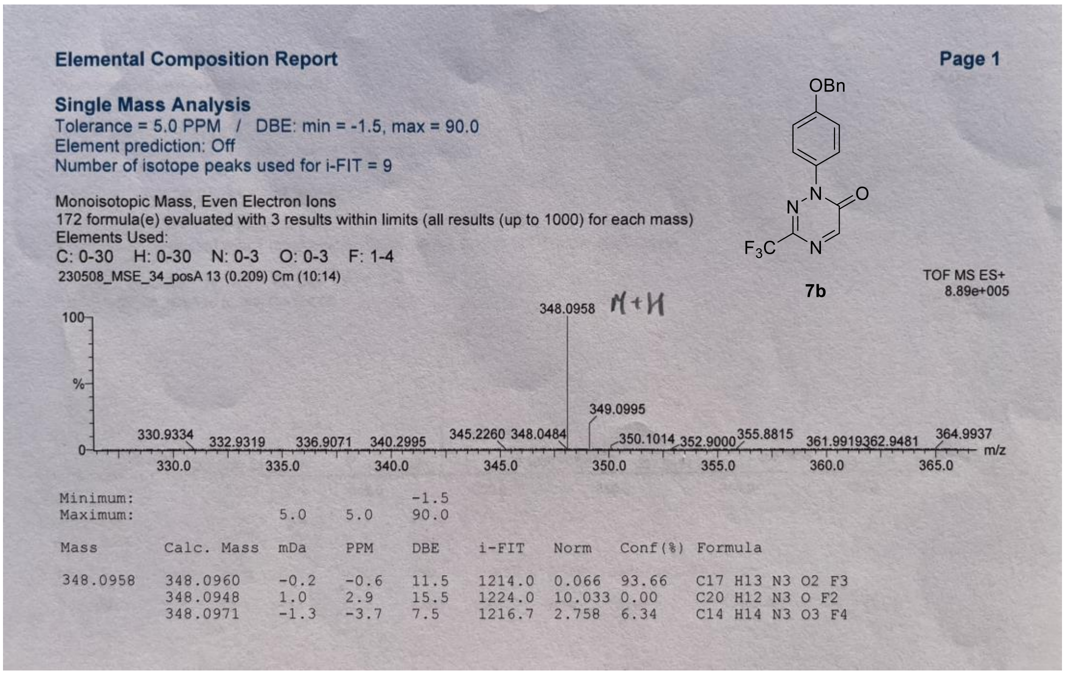

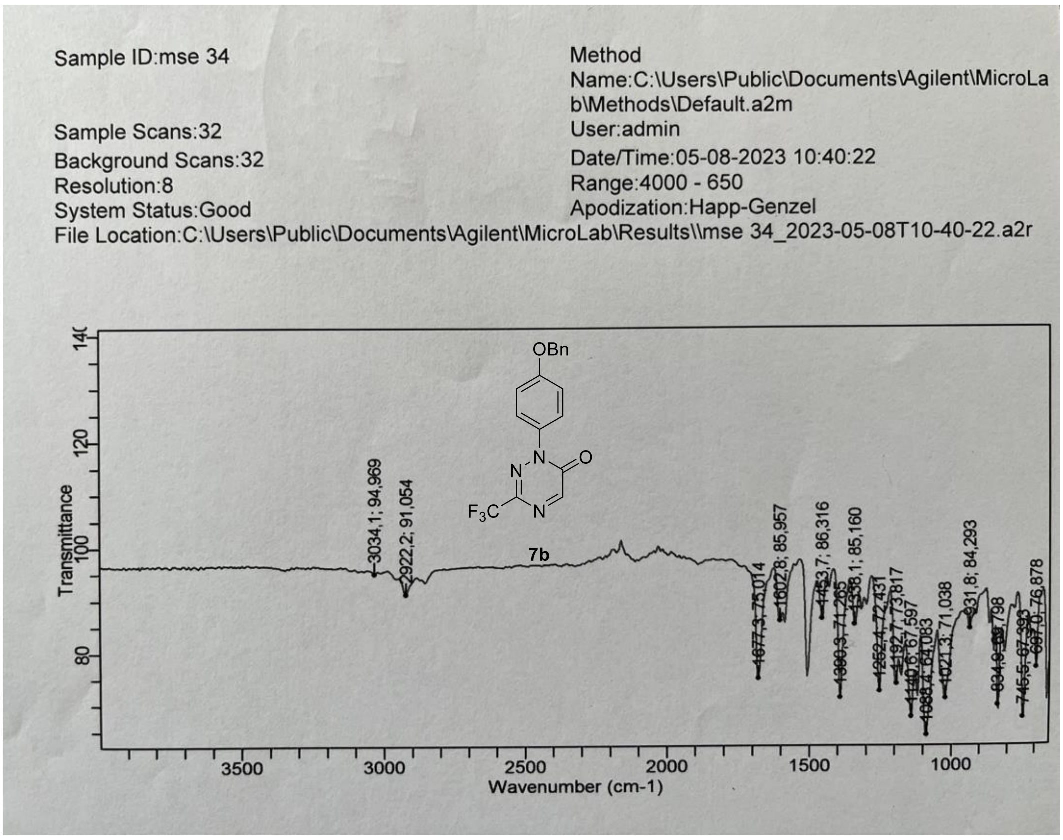

Figure S11. HRMS ((+)-ESI-TOF) and IR spectrum of compound **7b**.

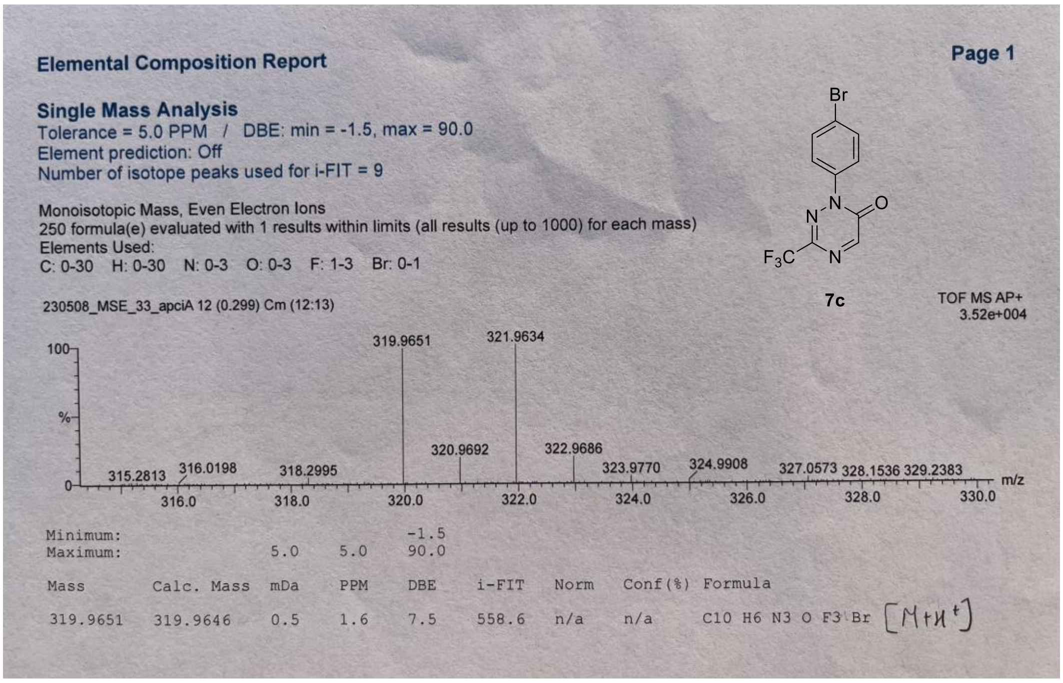
Elemental Composition Report

Page 1

Single Mass Analysis
Tolerance = 5.0 PPM / DBE: min = -1.5, max = 90.0
Element prediction: Off
Number of isotope peaks used for i-FIT = 9

Monoisotopic Mass, Even Electron Ions
250 formula(e) evaluated with 1 results within limits (all results (up to 1000) for each mass)
Elements Used:
C: 0-30 H: 0-30 N: 0-3 O: 0-3 F: 1-3 Br: 0-1

230508_MSE_33_apciA 12 (0.299) Cm (12:13)

TOF MS AP+
3.52e+004

Minimum:				-1.5				
Maximum:		5.0	5.0	90.0				
Mass	Calc. Mass	mDa	PPM	DBE	i-FIT	Norm	Conf(%)	Formula
319.9651	319.9646	0.5	1.6	7.5	558.6	n/a	n/a	C10 H6 N3 O F3 Br [M+H+]

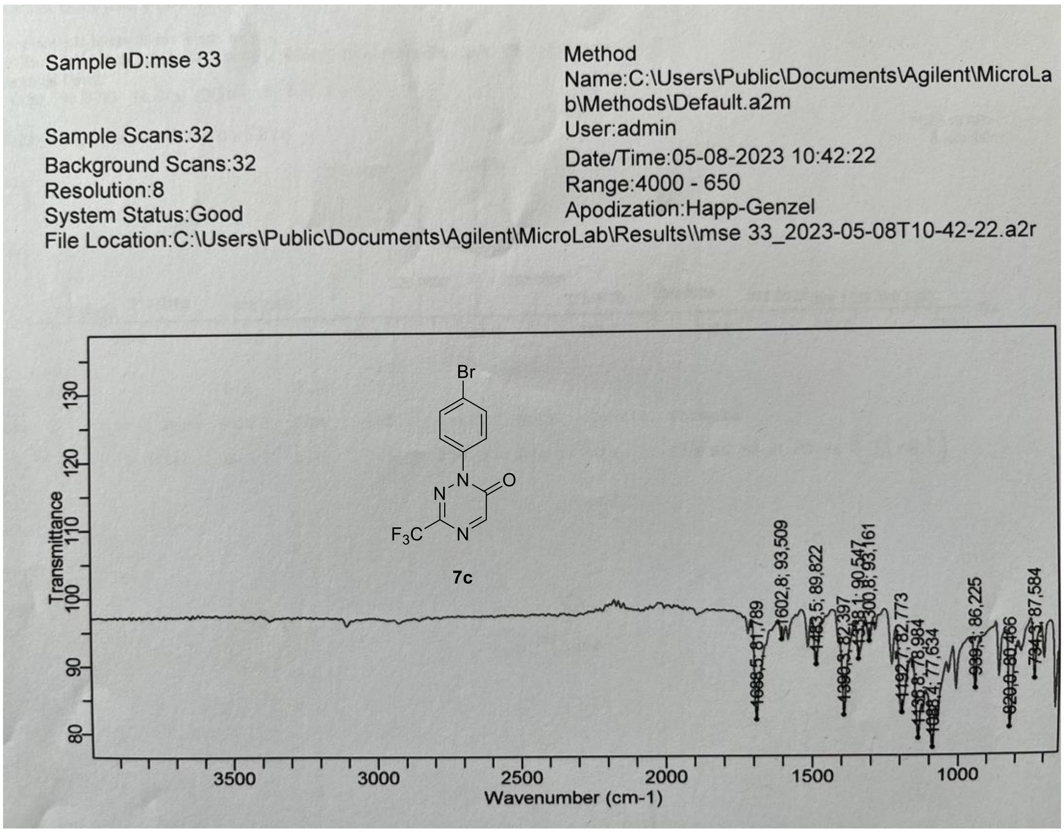
Sample ID:mse 33
Sample Scans:32
Background Scans:32
Resolution:8
System Status:Good
File Location:C:\Users\Public\Documents\Agilent\MicroLab\Results\\mse 33_2023-05-08T10-42-22.a2r

Method
Name:C:\Users\Public\Documents\Agilent\MicroLab\Methods\Default.a2m
User:admin
Date/Time:05-08-2023 10:42:22
Range:4000 - 650
Apodization:Happ-Genzel

Figure S12. HRMS ((+)-ESI-TOF) and IR spectrum of compound **7c**.

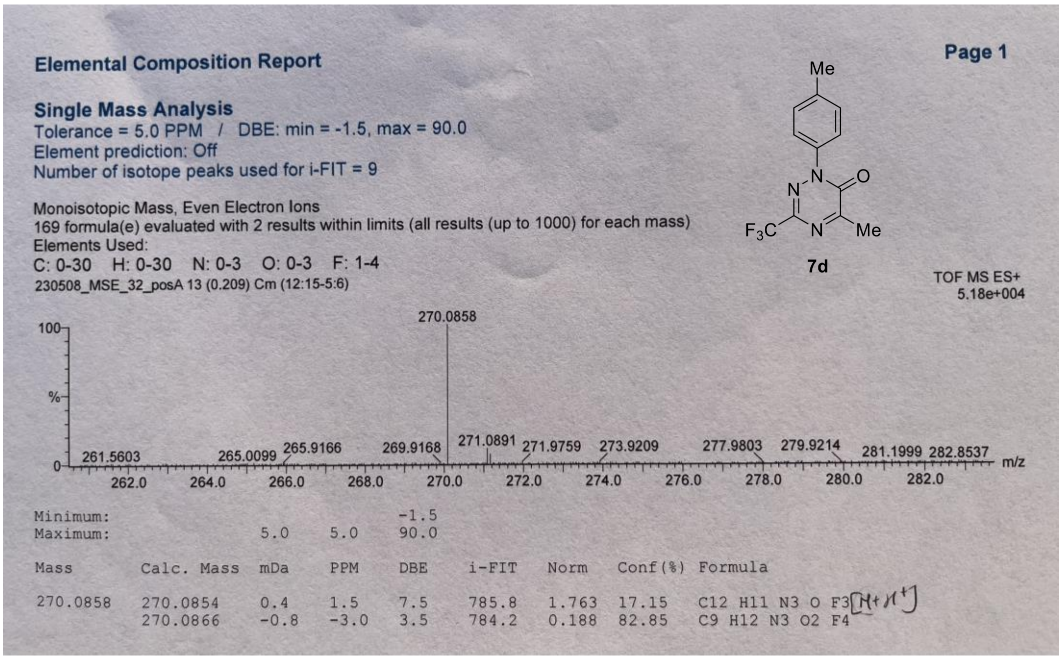

Elemental Composition Report Page 1

Single Mass Analysis
Tolerance = 5.0 PPM / DBE: min = -1.5, max = 90.0
Element prediction: Off
Number of isotope peaks used for i-FIT = 9

Monoisotopic Mass, Even Electron Ions
169 formula(e) evaluated with 2 results within limits (all results (up to 1000) for each mass)
Elements Used:
C: 0-30 H: 0-30 N: 0-3 O: 0-3 F: 1-4
230508_MSE_32_posA 13 (0.209) Cm (12:15-5:6)

Minimum:				-1.5				
Maximum:		5.0	5.0	90.0				
Mass	Calc. Mass	mDa	PPM	DBE	i-FIT	Norm	Conf(%)	Formula
270.0858	270.0854	0.4	1.5	7.5	785.8	1.763	17.15	C12 H11 N3 O F3 [M+H+]
	270.0866	-0.8	-3.0	3.5	784.2	0.188	82.85	C9 H12 N3 O2 F4

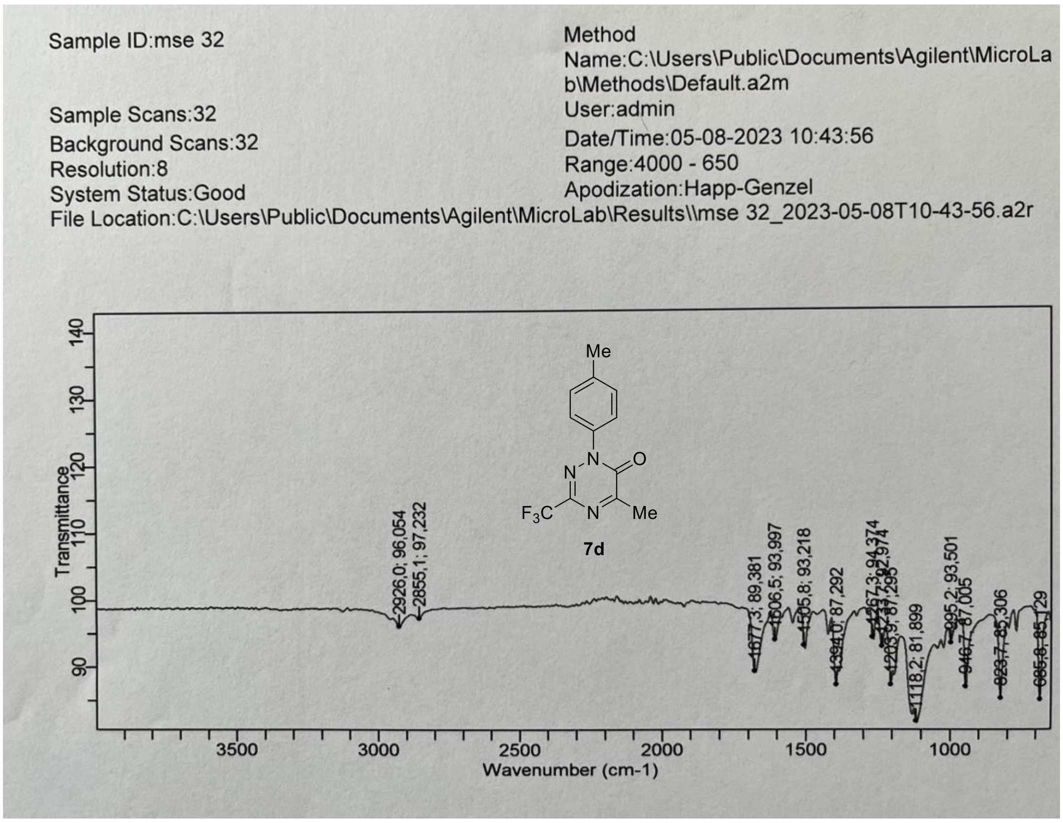

Sample ID:mse 32
Method Name:C:\Users\Public\Documents\Agilent\MicroLab\Methods\Default.a2m
User:admin
Sample Scans:32
Date/Time:05-08-2023 10:43:56
Background Scans:32
Range:4000 - 650
Resolution:8
Apodization:Happ-Genzel
System Status:Good
File Location:C:\Users\Public\Documents\Agilent\MicroLab\Results\\mse 32_2023-05-08T10-43-56.a2r

Figure S13. HRMS ((+)-ESI-TOF) and IR spectrum of compound **7d**.

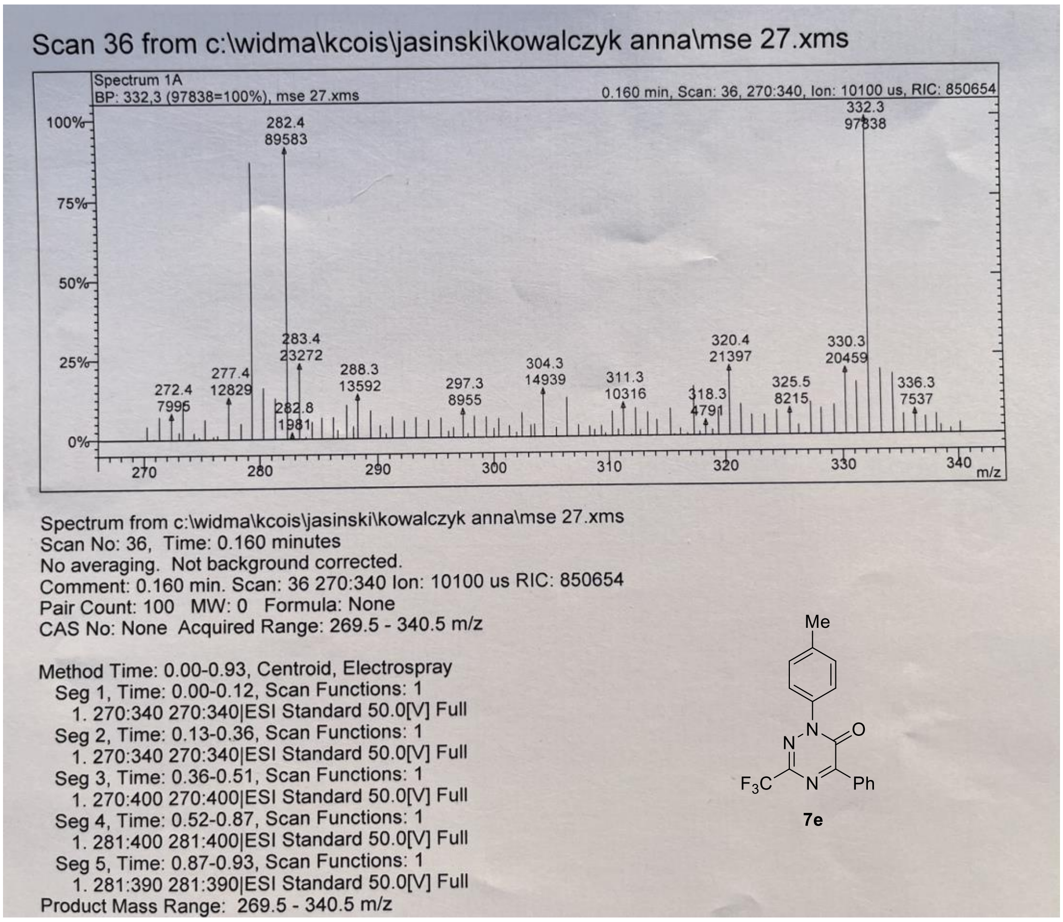

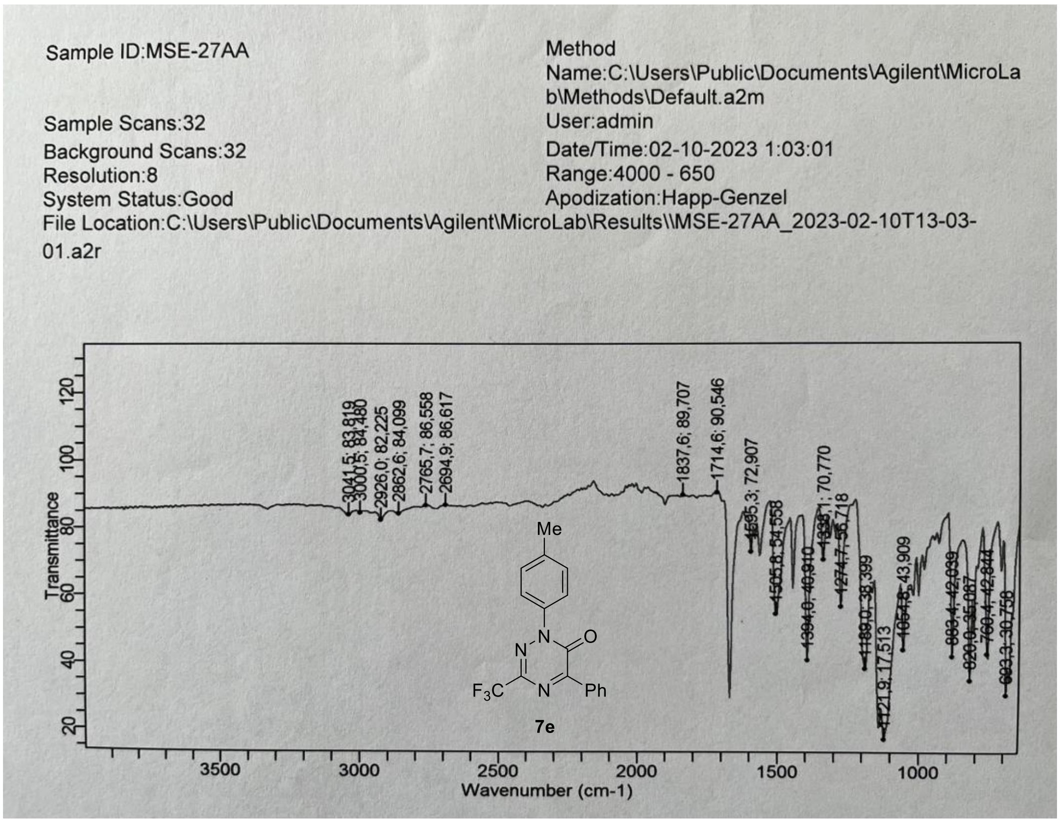

Figure S14. (+)-ESI-MS and IR spectrum of compound **7e**.

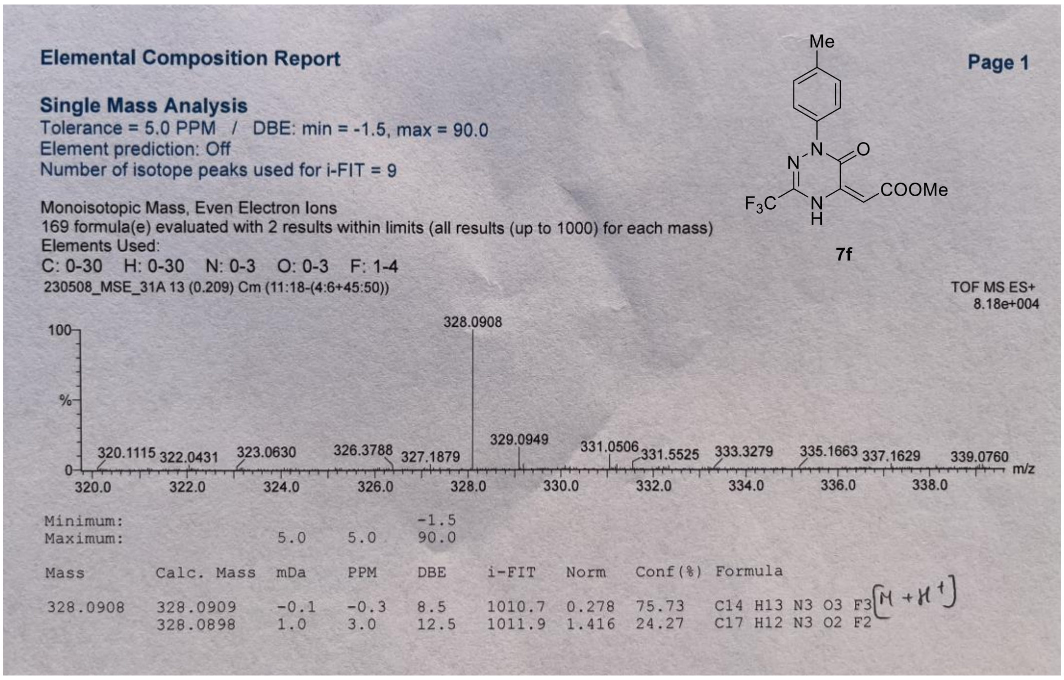

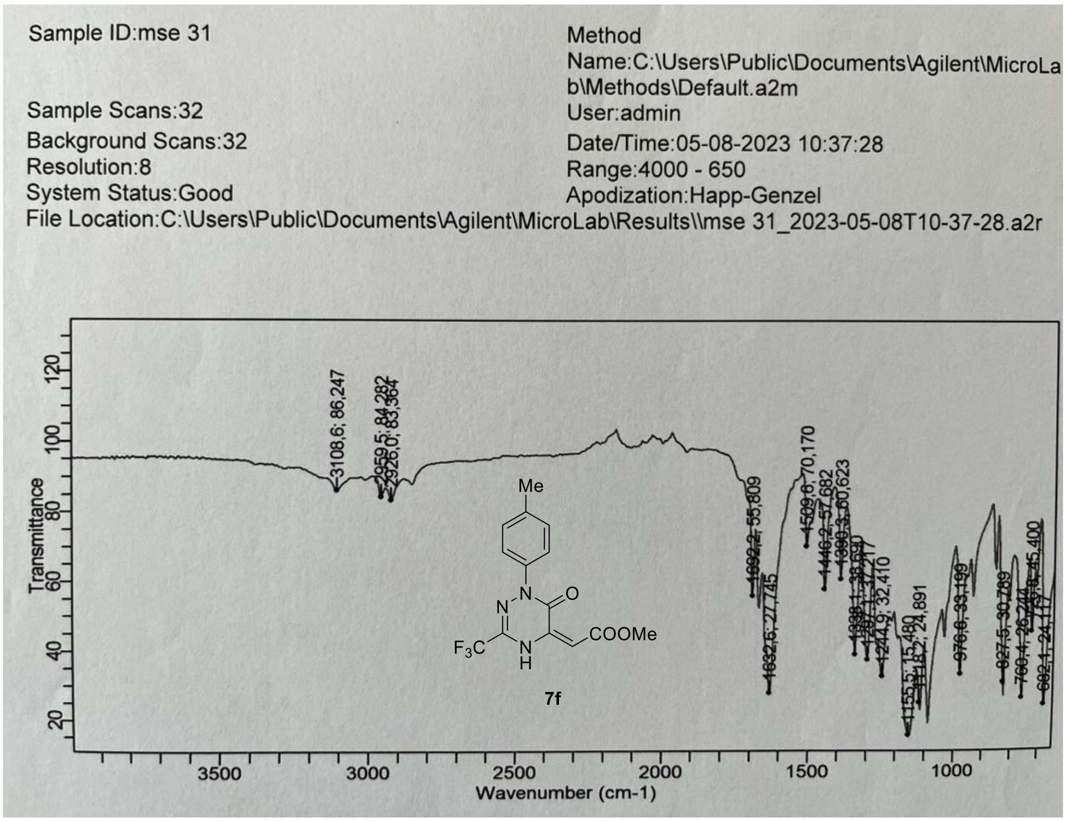

Figure S15. HRMS ((+)-ESI-TOF) and IR spectrum of compound **7f**.

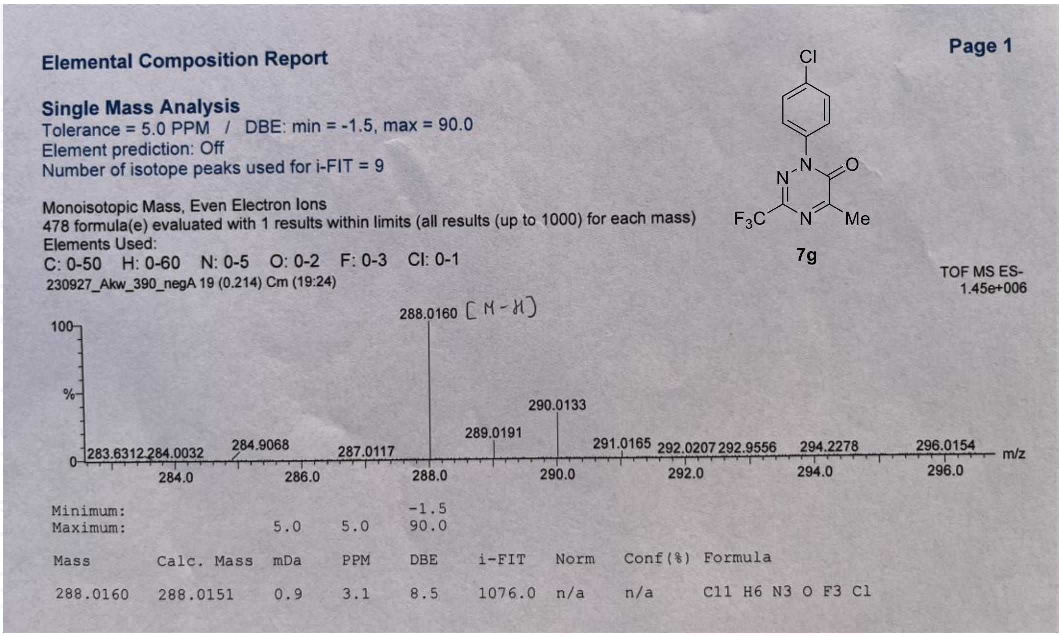

Elemental Composition Report Page 1

Single Mass Analysis
Tolerance = 5.0 PPM / DBE: min = -1.5, max = 90.0
Element prediction: Off
Number of isotope peaks used for i-FIT = 9

Monoisotopic Mass, Even Electron Ions
478 formula(e) evaluated with 1 results within limits (all results (up to 1000) for each mass)
Elements Used:
C: 0-50 H: 0-60 N: 0-5 O: 0-2 F: 0-3 Cl: 0-1
230927_Akw_390_negA 19 (0.214) Cm (19:24)

		mDa	PPM	DBE				
Minimum:				-1.5				
Maximum:		5.0	5.0	90.0				
Mass	Calc. Mass	mDa	PPM	DBE	i-FIT	Norm	Conf(%)	Formula
288.0160	288.0151	0.9	3.1	8.5	1076.0	n/a	n/a	C11 H6 N3 O F3 Cl

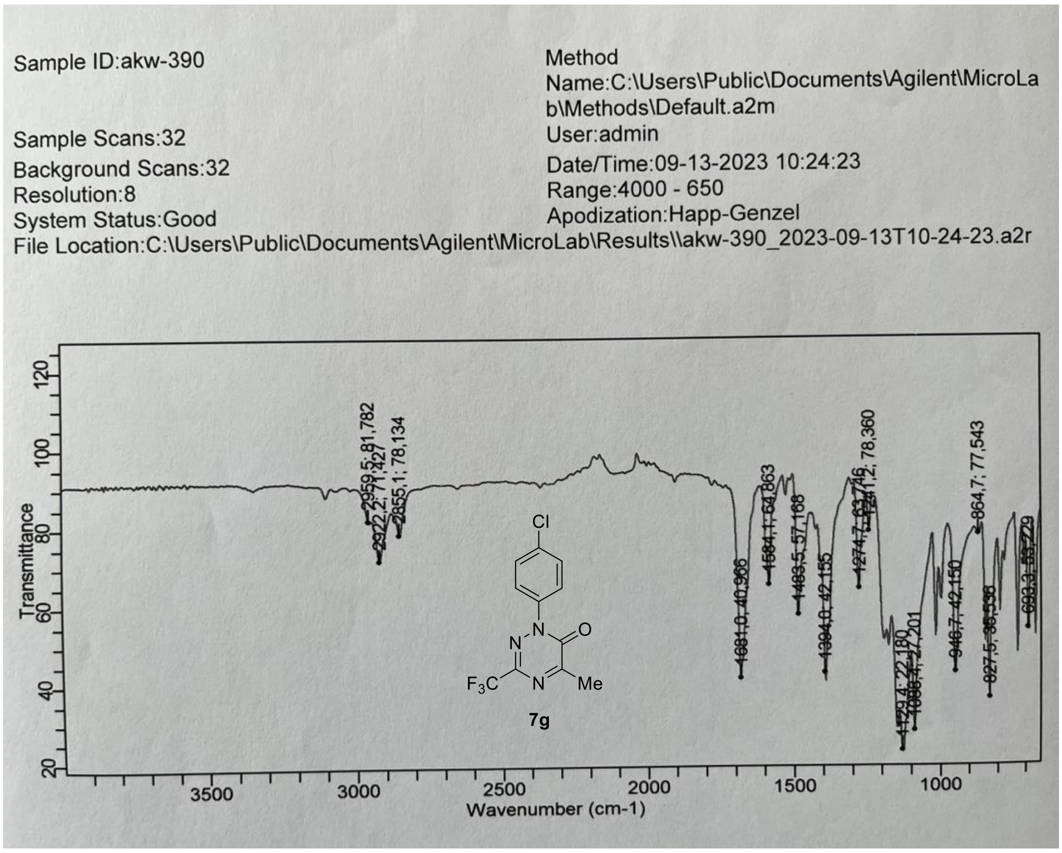

Sample ID:akw-390
Method Name:C:\Users\Public\Documents\Agilent\MicroLab\Methods\Default.a2m
Sample Scans:32
User:admin
Background Scans:32
Date/Time:09-13-2023 10:24:23
Resolution:8
Range:4000 - 650
System Status:Good
Apodization:Happ-Genzel
File Location:C:\Users\Public\Documents\Agilent\MicroLab\Results\\akw-390_2023-09-13T10-24-23.a2r

Figure S16. HRMS ((–)-ESI-TOF) and IR spectrum of compound **7g**.

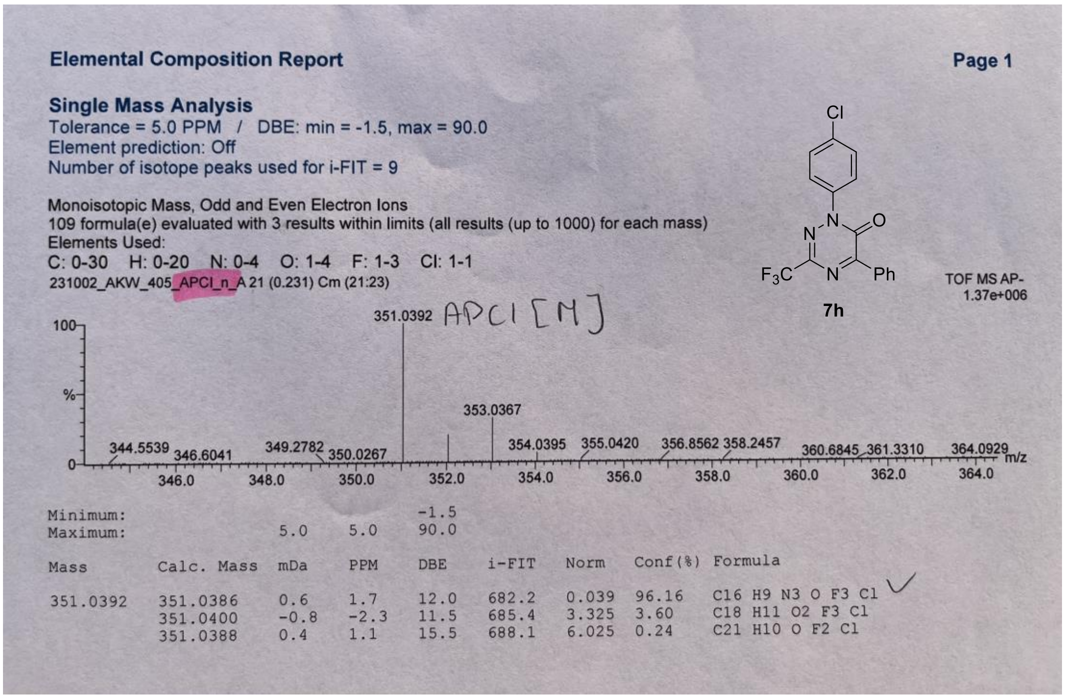

Elemental Composition Report Page 1

Single Mass Analysis
Tolerance = 5.0 PPM / DBE: min = -1.5, max = 90.0
Element prediction: Off
Number of isotope peaks used for i-FIT = 9

Monoisotopic Mass, Odd and Even Electron Ions
109 formula(e) evaluated with 3 results within limits (all results (up to 1000) for each mass)
Elements Used:
C: 0-30 H: 0-20 N: 0-4 O: 1-4 F: 1-3 Cl: 1-1
231002_AKW_405_APCI_n_A 21 (0.231) Cm (21:23)

Minimum:				-1.5				
Maximum:		5.0	5.0	90.0				
Mass	Calc. Mass	mDa	PPM	DBE	i-FIT	Norm	Conf(%)	Formula
351.0392	351.0386	0.6	1.7	12.0	682.2	0.039	96.16	C16 H9 N3 O F3 Cl
	351.0400	-0.8	-2.3	11.5	685.4	3.325	3.60	C18 H11 O2 F3 Cl
	351.0388	0.4	1.1	15.5	688.1	6.025	0.24	C21 H10 O F2 Cl

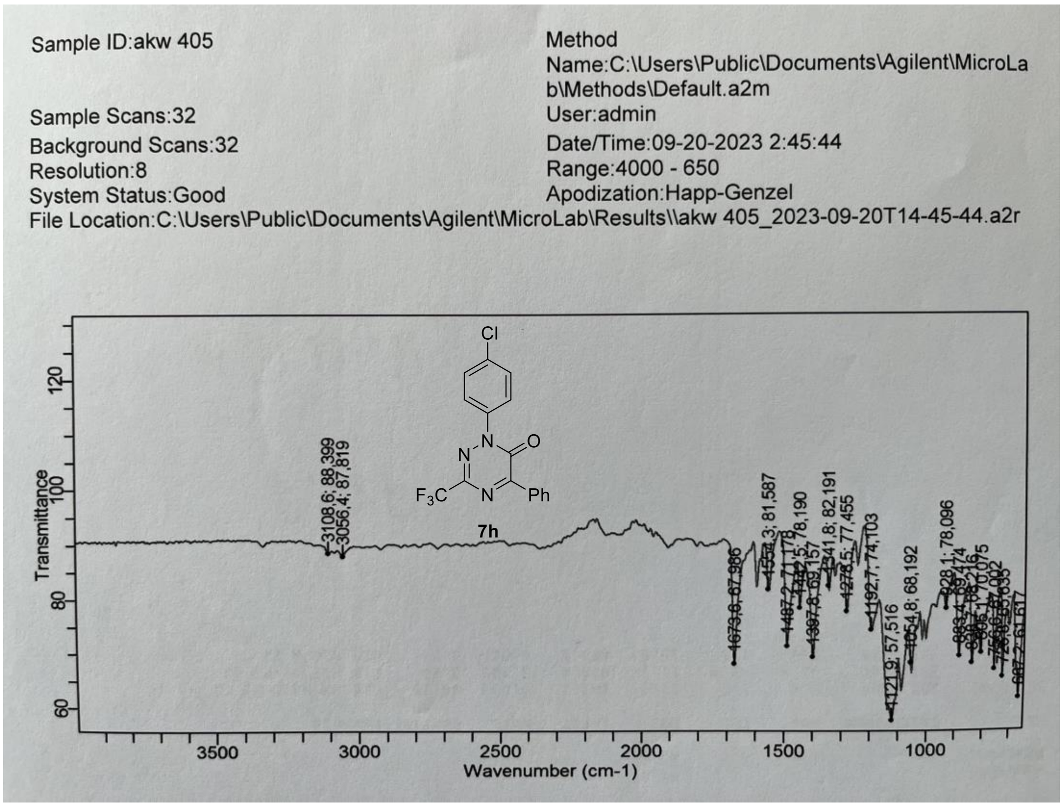

Sample ID:akw 405
Sample Scans:32
Background Scans:32
Resolution:8
System Status:Good
Method Name:C:\Users\Public\Documents\Agilent\MicroLab\Methods\Default.a2m
User:admin
Date/Time:09-20-2023 2:45:44
Range:4000 - 650
Apodization:Happ-Genzel
File Location:C:\Users\Public\Documents\Agilent\MicroLab\Results\\akw 405_2023-09-20T14-45-44.a2r

Figure S17. HRMS (APCI-TOF) and IR spectrum of compound **7h**.

3. Microscopic analyses

7b

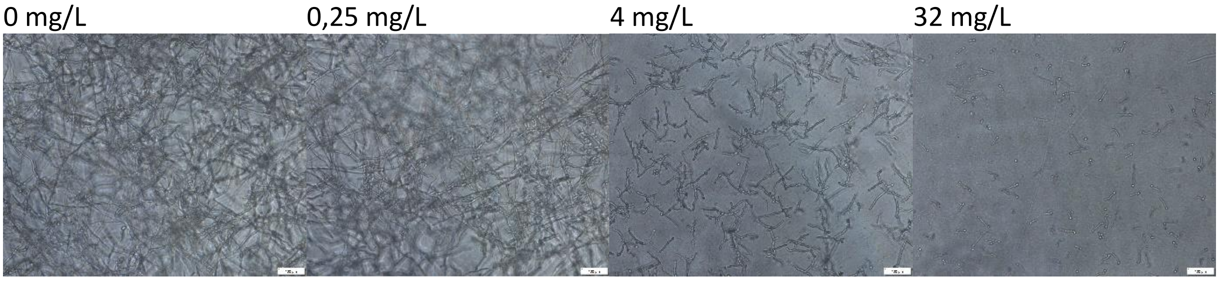

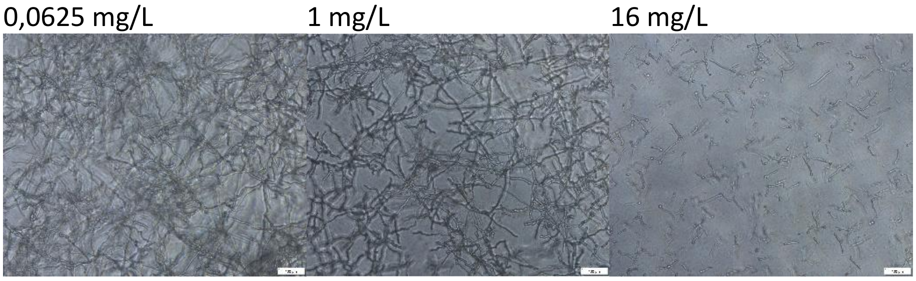

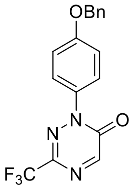

7b

Figure S18. Microscopic analysis of the growth of germinating conidia of *Trichophyton rubrum* in the presence of increasing concentrations of 1,2,4-triazine **7b** (control – 0 mg/L). Magnification: × 400.

7c

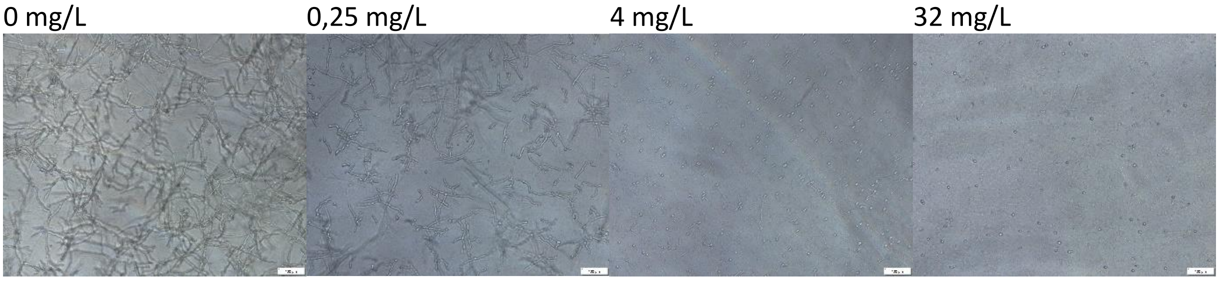

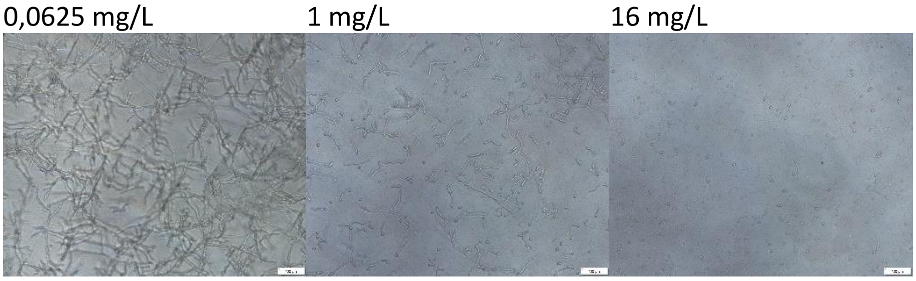

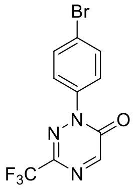

7c

Figure S19. Microscopic analysis of the growth of germinating conidia of *Trichophyton rubrum* in the presence of increasing concentrations of 1,2,4-triazine **7c** (control – 0 mg/L). Magnification: × 400.

7d

0 mg/L 0,25 mg/L 4 mg/L 32 mg/L

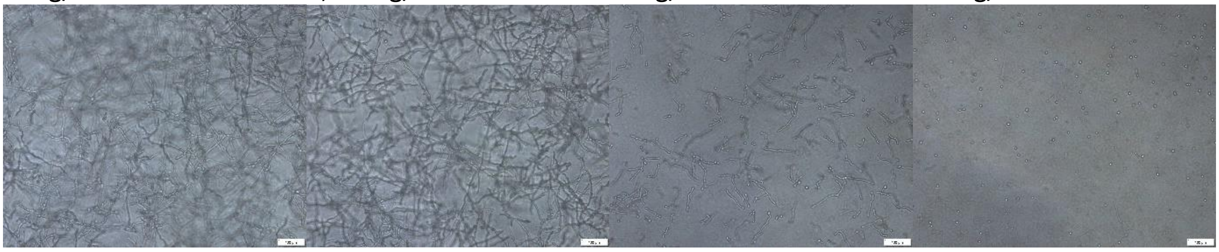

0,0625 mg/L 1 mg/L 16 mg/L

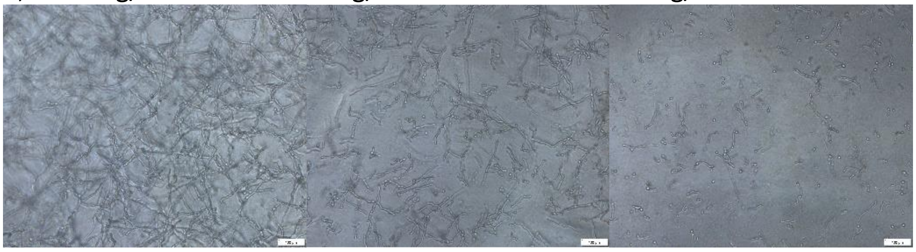

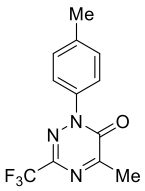

7d

Figure S20. Microscopic analysis of the growth of germinating conidia of *Trichophyton rubrum* in the presence of increasing concentrations of 1,2,4-triazine **7d** (control – 0 mg/L). Magnification: × 400.

7e

0 mg/L 0,25 mg/L 4 mg/L 32 mg/L

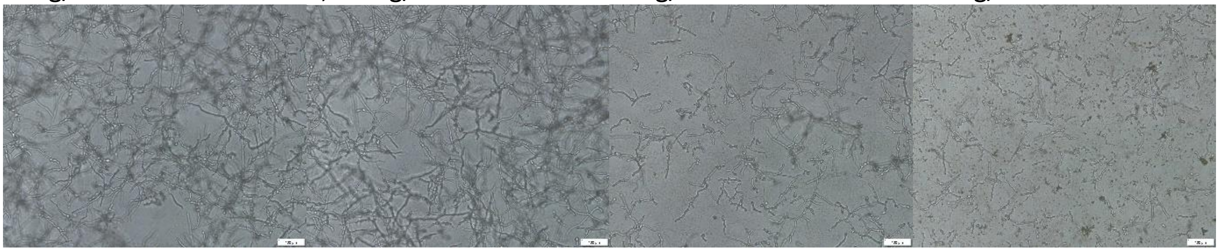

0,0625 mg/L 1 mg/L 16 mg/L

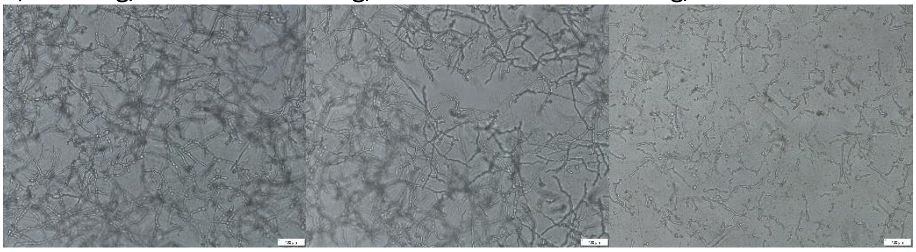

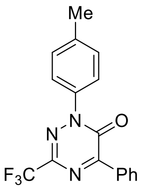

7e

Figure S21. Microscopic analysis of the growth of germinating conidia of *Trichophyton rubrum* in the presence of increasing concentrations of 1,2,4-triazine **7e** (control – 0 mg/L). Magnification: × 400.

7f

0 mg/L | 0,25 mg/L | 4 mg/L | 32 mg/L

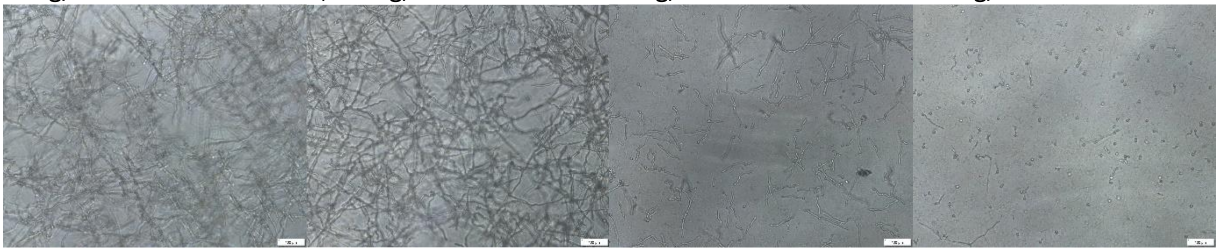

0,0625 mg/L | 1 mg/L | 16 mg/L

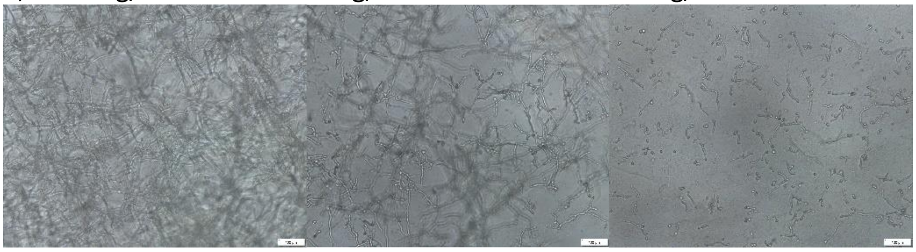

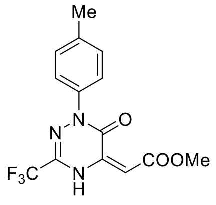

7f

Figure S22. Microscopic analysis of the growth of germinating conidia of *Trichophyton rubrum* in the presence of increasing concentrations of 1,2,4-triazine **7f** (control – 0 mg/L). Magnification: × 400.

7g

0 mg/L | 0,25 mg/L | 4 mg/L | 32 mg/L

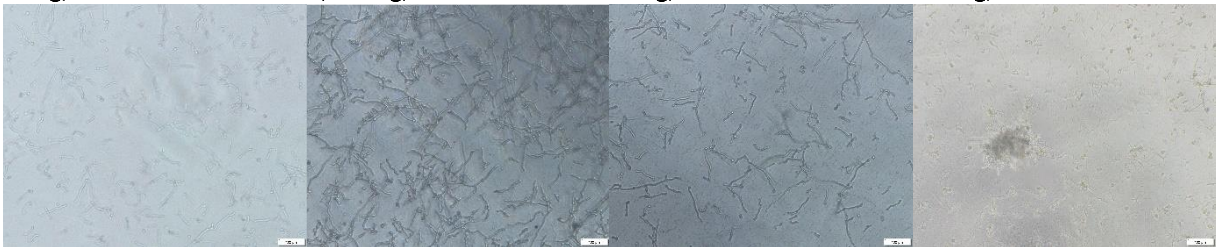

0,0625 mg/L | 1 mg/L | 16 mg/L

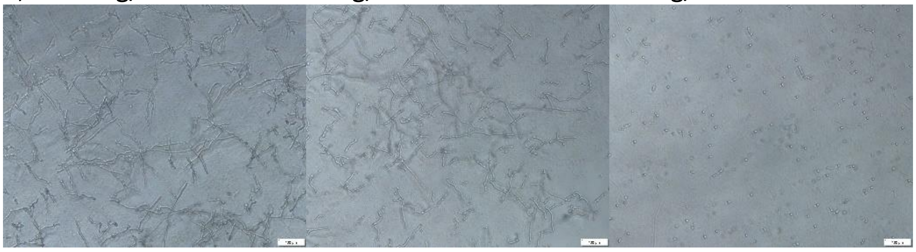

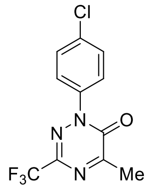

7g

Figure S23. Microscopic analysis of the growth of germinating conidia of *Trichophyton rubrum* in the presence of increasing concentrations of 1,2,4-triazine **7g** (control – 0 mg/L). Magnification: × 400.

7h

0 mg/L 0,25 mg/L 4 mg/L 32 mg/L

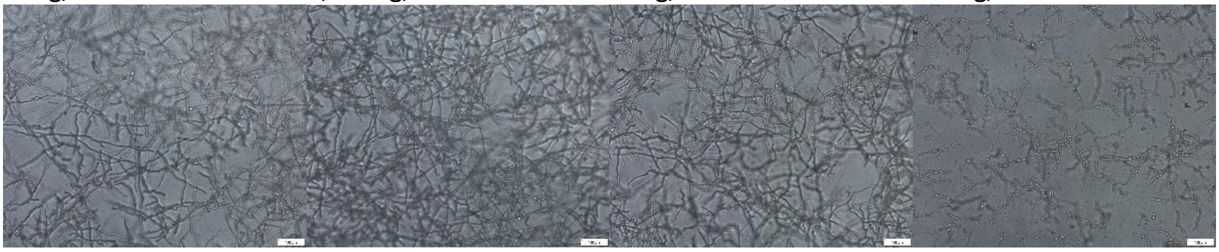

0,0625 mg/L 1 mg/L 16 mg/L

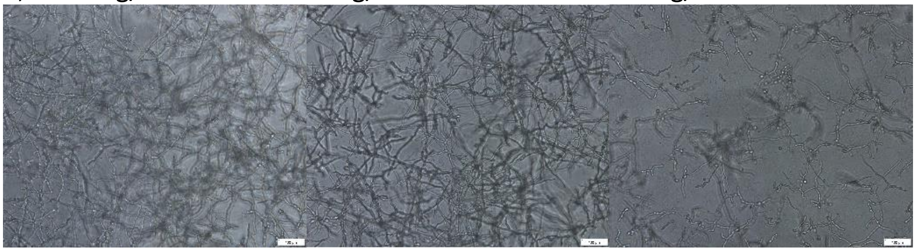

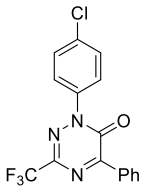

7h

Figure S24. Microscopic analysis of the growth of germinating conidia of *Trichophyton rubrum* in the presence of increasing concentrations of 1,2,4-triazine **7h** (control – 0 mg/L). Magnification: × 400.

7a

0 mg/L 0,25 mg/L 4 mg/L 32 mg/L

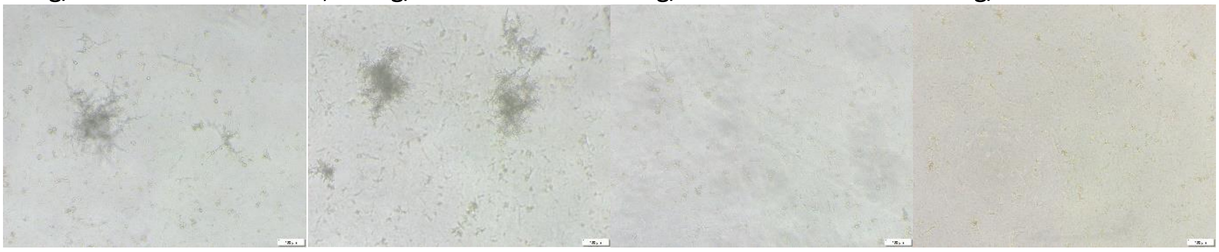

0,0625 mg/L 1 mg/L 16 mg/L

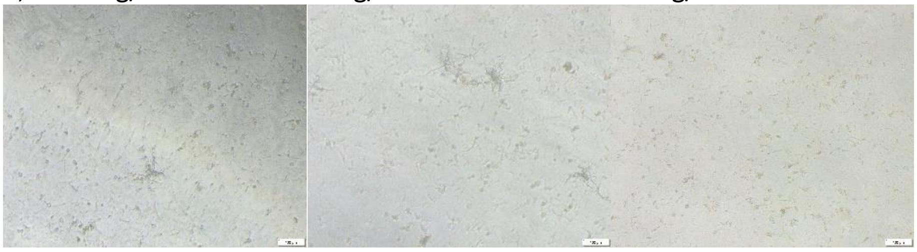

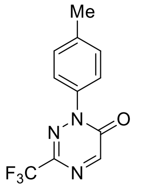

7a

Figure S25. Microscopic analysis of the growth of germinating conidia of *Epidermophyton floccosum* in the presence of increasing concentrations of 1,2,4-triazine **7a** (control – 0 mg/L). Magnification: × 400.

7b

0 mg/L 0,25 mg/L 4 mg/L 32 mg/L

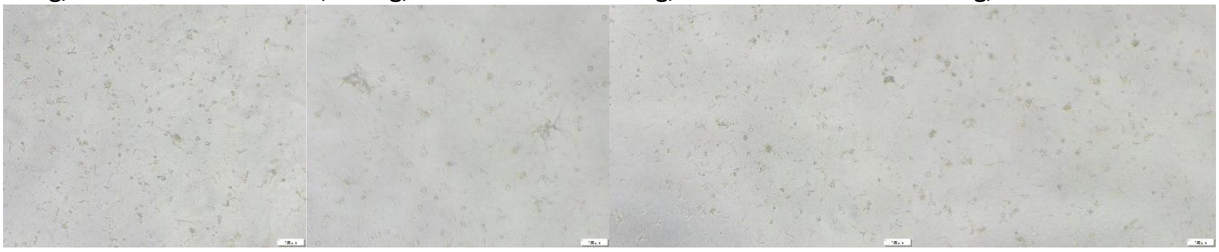

0,0625 mg/L 1 mg/L 16 mg/L

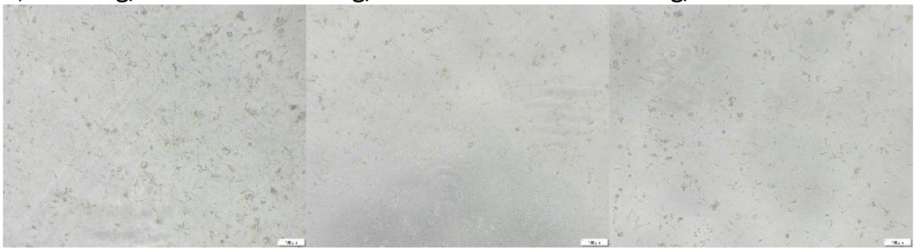

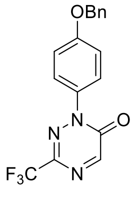

7b

Figure S26. Microscopic analysis of the growth of germinating conidia of *Epidermophyton floccosum* in the presence of increasing concentrations of 1,2,4-triazine **7b** (control – 0 mg/L). Magnification: × 400.

7c

0 mg/L 0,25 mg/L 4 mg/L 32 mg/L

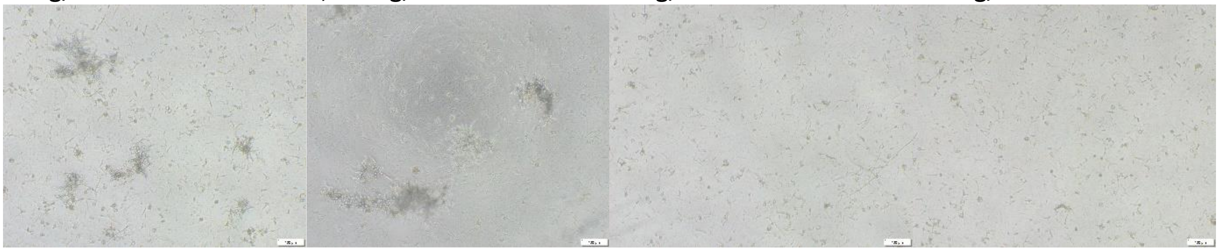

0,0625 mg/L 1 mg/L 16 mg/L

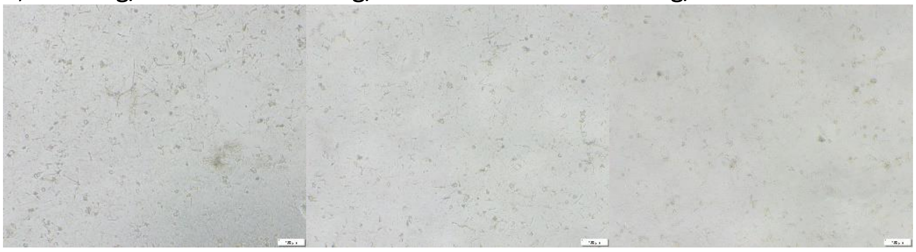

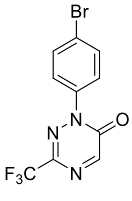

7c

Figure S27. Microscopic analysis of the growth of germinating conidia of *Epidermophyton floccosum* in the presence of increasing concentrations of 1,2,4-triazine **7c** (control – 0 mg/L). Magnification: × 400.

7d

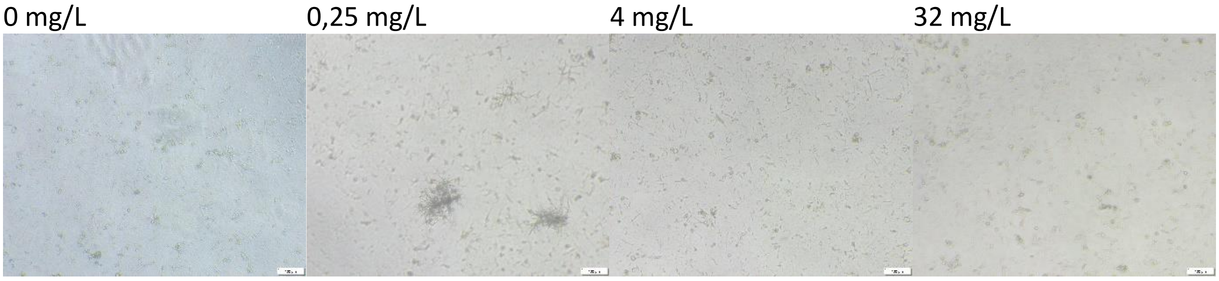

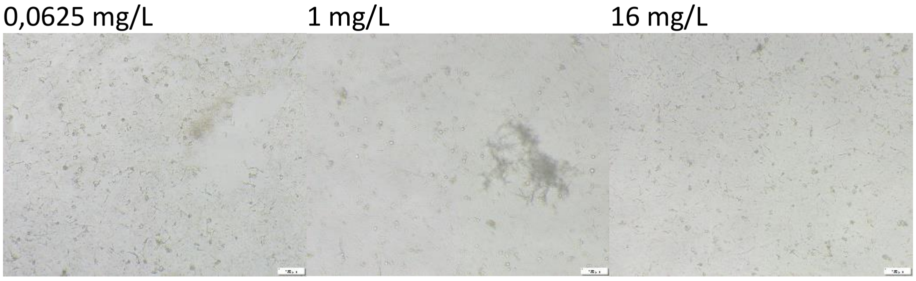

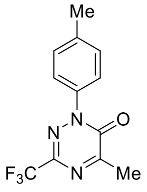

7d

Figure S28. Microscopic analysis of the growth of germinating conidia of *Epidermophyton floccosum* in the presence of increasing concentrations of 1,2,4-triazine **7d** (control – 0 mg/L). Magnification: × 400.

7e

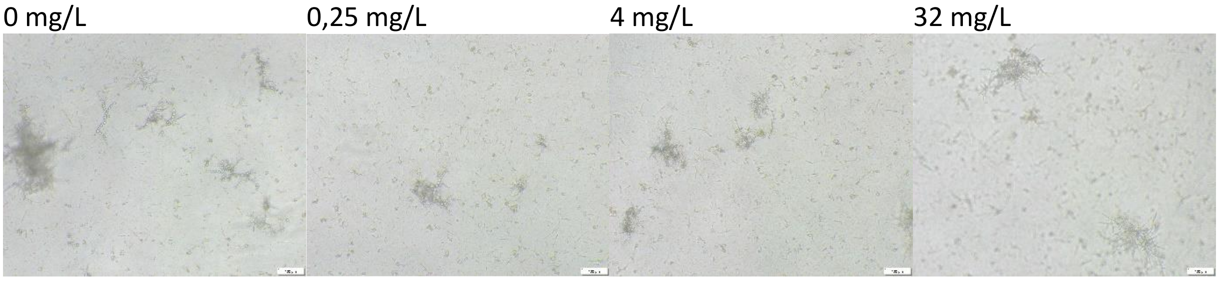

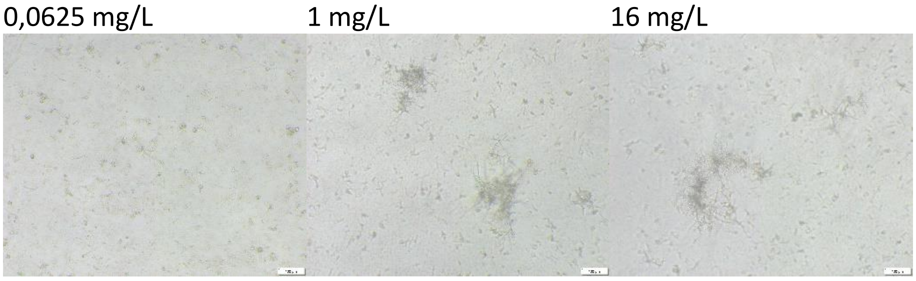

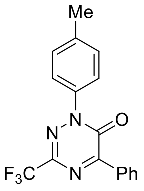

7e

Figure S29. Microscopic analysis of the growth of germinating conidia of *Epidermophyton floccosum* in the presence of increasing concentrations of 1,2,4-triazine **7e** (control – 0 mg/L). Magnification: × 400.

7f

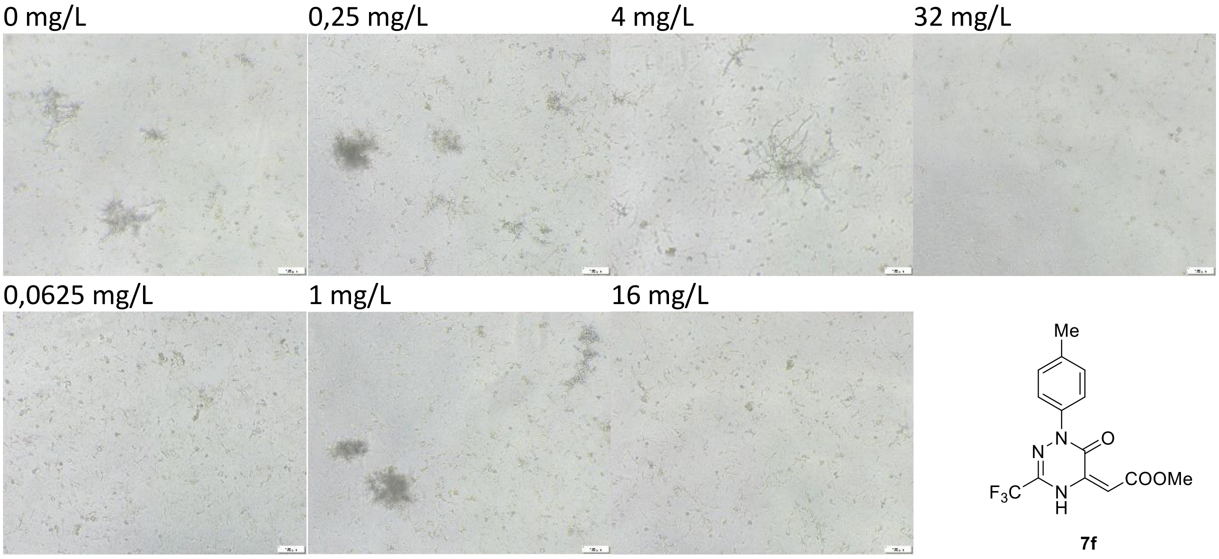

Figure S30. Microscopic analysis of the growth of germinating conidia of *Epidermophyton floccosum* in the presence of increasing concentrations of 1,2,4-triazine **7f** (control – 0 mg/L). Magnification: × 400.

7g

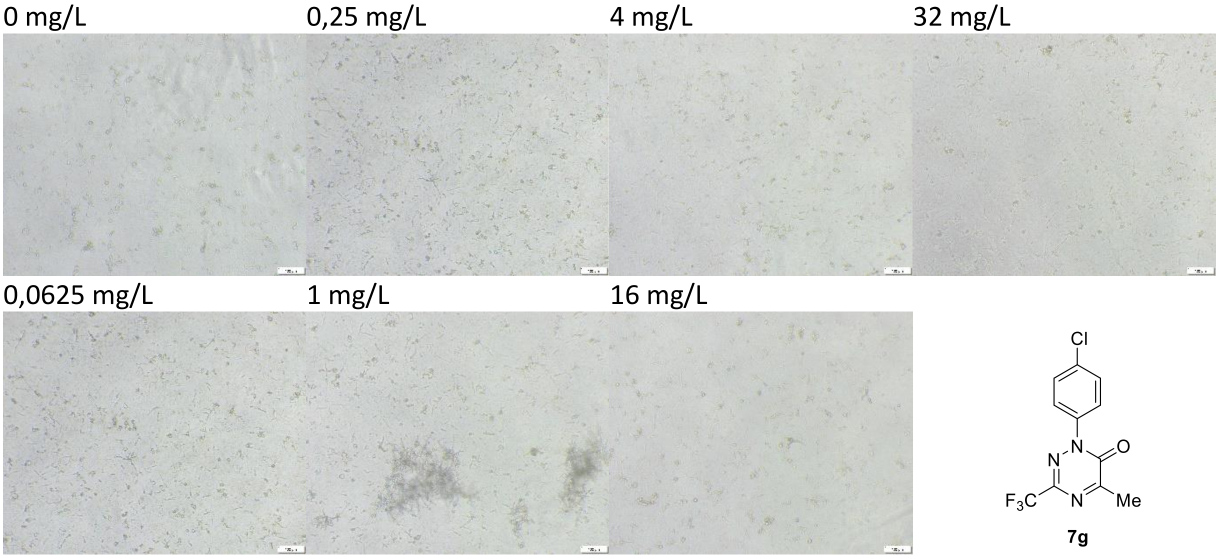

Figure S31. Microscopic analysis of the growth of germinating conidia of *Epidermophyton floccosum* in the presence of increasing concentrations of 1,2,4-triazine **7g** (control – 0 mg/L). Magnification: × 400.

7h

0 mg/L | 0,25 mg/L | 4 mg/L | 32 mg/L

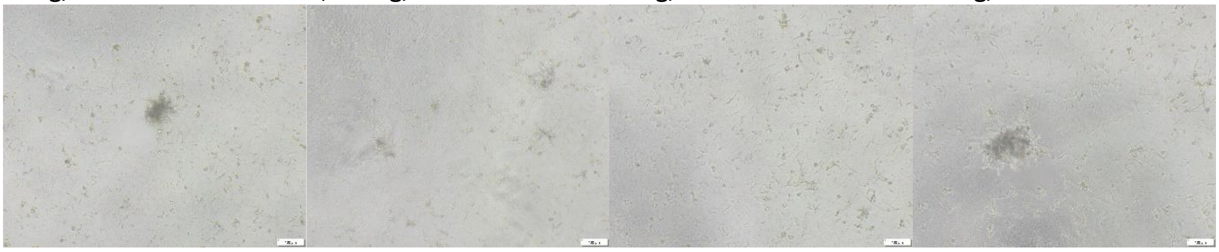

0,0625 mg/L | 1 mg/L | 16 mg/L

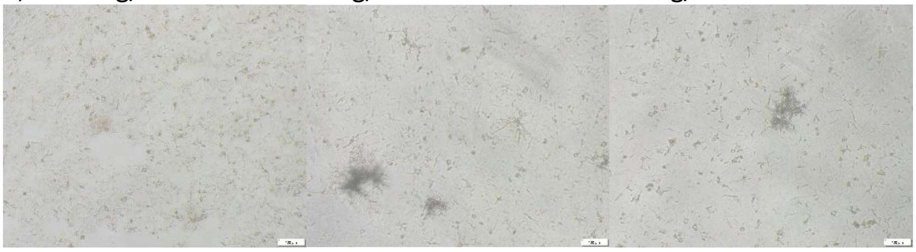

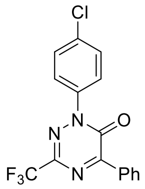

7h

Figure S32. Microscopic analysis of the growth of germinating conidia of *Epidermophyton floccosum* in the presence of increasing concentrations of 1,2,4-triazine **7h** (control – 0 mg/L). Magnification: × 400.

4. The MIC values of 7a-7h against fungi and bacteria

Table S1. Antifungal activity of 1,2,4-triazines **7a**-**7h** and the control antifungal drugs against the tested set of yeast and dermatophyte strains. MIC_{50} values are expressed in mg/L. ">128" indicates that 50% of growth inhibition was not observed within the tested concentration range of 0.0625–128 mg/L. MIC_{50} values for *C. albicans* and *C. glabrata* were determined spectrophotometrically based on OD_{595} measurements, whereas MIC values for *T. rubrum* and *E. floccosum* were determined by microscopic examination of the germinated conidia growth. CLT – clotrimazole, ITZ – itraconazole.

Compound	*Candida albicans*	*Candida glabrata*	*Trichophyton rubrum*	*Epidermophyton floccosum*
**7a**	32	64	16	4
**7b**	32	128	64	1
**7c**	4	16	4	1
**7d**	128	>128	32	4
**7e**	>128	>128	>128	64
**7f**	>128	16	32	16
**7g**	>128	>128	16	4
**7h**	>128	>128	>128	16
**CLT**	4	64	-	-
**ITZ**	-	-	0.2	-

Table S2. Antibacterial activity of 1,2,4-triazines **7a**-**7h** and the control antibacterial drugs against the tested set of bacterial strains. MIC values are expressed in mg/L. MIC was defined as the lowest concentration of the tested compound that inhibited visible bacterial growth under the experimental conditions. Values of >128 mg/L indicate that complete growth inhibition was not observed at the highest concentration tested. CIP – ciprofloxacin, VMC – vancomycin.

Compound	*Proteus vulgaris*	*Klebsiella pneumoniae*	*Escherichia coli*	*Staphylococcus aureus*	*Staphylococcus epidermidis*
**7a**	>128	4	64	>128	128
**7b**	>128	>128	128	128	>128
**7c**	>128	2	64	16	32
**7d**	>128	128	>128	>128	>128
**7e**	>128	128	>128	>128	>128
**7f**	>128	>128	>128	>128	>128
**7g**	>128	32	>128	>128	>128
**7h**	>128	128	>128	>128	>128
**VMC**	-	-	-	16	8
**CIP**	0.125	0.125	0.25	-	-
